# Variation in the *AvrSr35* gene determines *Sr35* resistance against wheat stem rust race Ug99

**DOI:** 10.1126/science.aao7294

**Published:** 2017-12-22

**Authors:** Andres Salcedo, William Rutter, Shichen Wang, Alina Akhunova, Stephen Bolus, Shiaoman Chao, Nickolas Anderson, Monica Fernandez De Soto, Matthew Rouse, Les Szabo, Robert L. Bowden, Jorge Dubcovsky, Eduard Akhunov

**Affiliations:** 1 Department of Plant Pathology, Kansas State University, Manhattan, KS 66506, USA; 2 Integrated Genomics Facility, Kansas State University, Manhattan, KS 66506, USA; 3 Department of Plant Sciences, University of California, Davis, CA 95616, USA; 4 U.S. Department of Agriculture, Agricultural Research Service (USDA-ARS) Cereal Crops Research Unit, Fargo, ND 58102, USA; 5 USDA-ARS, Cereal Disease Laboratory, St. Paul, MN 55108, USA; 6 Department of Plant Pathology, University of Minnesota, St. Paul, MN 55108, USA; 7 USDA-ARS, Hard Winter Wheat Genetics Research Unit, Manhattan, KS 66506, USA; 8 Howard Hughes Medical Institute (HHMI), Chevy Chase, MD 20815, USA

## Abstract

*Puccinia graminis* f. sp. *tritici* (*Pgt*) causes wheat stem rust, a devastating fungal disease. The *Sr35* resistance gene confers immunity against this pathogen’s most virulent races, including Ug99. We used comparative whole-genome sequencing of chemically mutagenized and natural *Pgt* isolates to identify a fungal gene named *AvrSr35* that is required for *Sr35* avirulence. The *AvrSr35* gene encodes a secreted protein capable of interacting with Sr35 and triggering the immune response. We show that the origin of *Pgt* isolates virulent on *Sr35* is associated with the nonfunctionalization of the *AvrSr35* gene by the insertion of a mobile element. The discovery of *AvrSr35* provides a new tool for *Pgt* surveillance, identification of host susceptibility targets, and characterization of the molecular determinants of immunity in wheat.

**T**he emergence of new virulent races of pathogens that can overcome the resistance of existing crop cultivars poses a threat to global food security. A prime example is the outbreak of wheat stem rust in Africa that was caused by a broadly virulent *Puccinia graminis* f. sp. *tritici (Pgt)* race, Ug99, detected in Uganda in 1999 (*1*). Ug99 was virulent on most of the wheat varieties grown in Europe, Asia, and the United States, prompting research into the discovery of Ug99-effective resistance genes. Since the discovery of Ug99, *Pgt* surveillance identified new Ug99-derived strains virulent against additional wheat resistance genes (*2*).

Plant resistance genes *(R)* defend against an invading pathogen by detecting the corresponding pathogen avirulence factors (*Avr*), which are often secreted effector proteins. *R* genes encode receptors that trigger an immune response upon perception of pathogen *Avr* factors. This response results in localized cell death at the site of infection [hypersensitive response (HR)] (*3**–**5*). A pathogen lacking an *Avr* gene renders the corresponding plant *R* gene ineffective. Here, we identified the fungal *Avr* gene recognized by the Ug99-effective wheat stem rust resistance gene *Sr35* (*6*) and investigated the origin of *Sr35*-virulent fungal isolates.

Using confocal microscopy of *Pgt*-infected leaf tissues from resistant (*Sr35+*) and susceptible (*Sr35*–) wheat lines, we demonstrated that *Sr35* triggers a resistance response at the early stages of infection (Fig. 1) (*7*). In wheat lineU6169 (*Sr35*+), the development of fungal infection hyphae stopped even before the formation of a haustorium, the structurewithwhich the fungus extracts nutrients from its host plant. This early immune response is consistent with the lack of pronounced HR symptoms in *Triticum monococcum* accession G2919 used to identify the *Sr35* gene (*6*) and suggested early expression of a fungal gene recognized by *Sr35*. To identify this *Avr* gene, we mutagenized the spores of the *Pgt* race RKQQC (*Sr35*-avirulent isolate 99KS76A-1) with ethylmethane sulfonate (EMS). We isolated 15 *Pgt* mutants virulent to the *Sr35* gene, suggesting that they carry mutations affecting the *Sr35*-specific *Avr* factor (tables S1 and S2) (*7*). Both microscopy and timecourseRNA- sequencing (RNA-seq) analyses showed no obvious effects of these mutations on the *Pgt* mutants’ interaction with a wheat host compared to the wild-type *Pgt* isolate (figs. S1 and S2, and tables S3 to S5) (*7*), perhaps due to the functional redundancy of virulence factors that can compensatemutations in the *AvrSr35* gene (*8*). The genome of the wild-type *Pgt* isolate was assembled, annotated using RNA-seq data (tables S4 and S6), and compared with Illumina reads generated for each of 15 independent *Pgt* mutants (table S7), resulting in the detection of 30,429 EMS-induced mutations (table S8 and data S1).

**Fig. 1:**
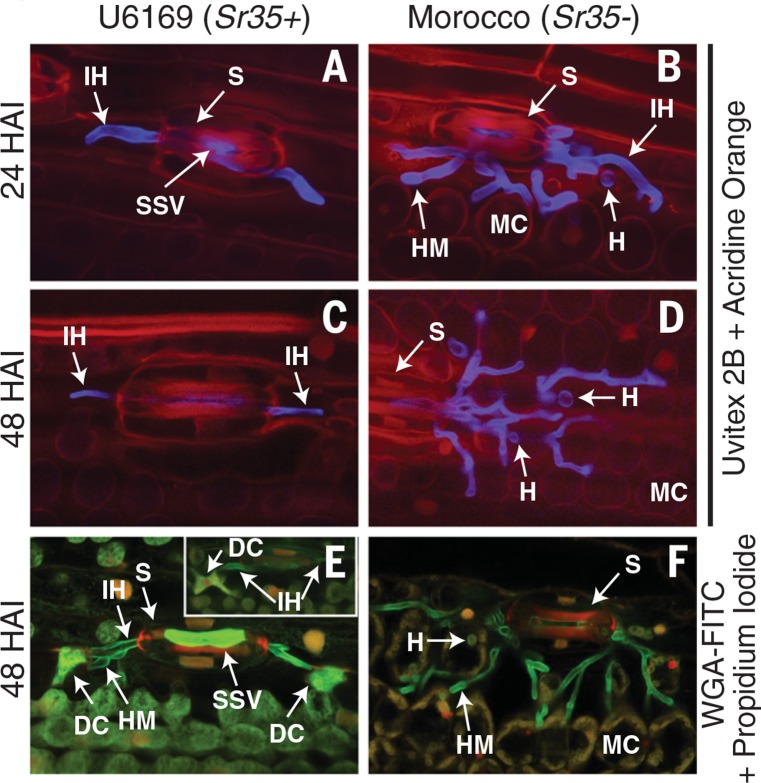
***Sr35* provides prehaustorial resistance against *Pgt*.** Infected leaves of susceptible cultivar Morocco (*Sr35–*) and resistant line U6169 (*Sr35+*) were collected 28 and 48 hours after infection (HAI). (**A** and **B**) Fungal infection hyphae (IH) (stained blue) entered the leaf mesophyll tissue (stained red) through the plant stoma (S) in both wheat lines at 24 HAI. Fungal haustoria (H) developed only in susceptible Morocco (B). (**C** and **D**) Imaging at 48 HAI revealed fungal growth in susceptible Morocco (D) but no further fungal growth in U6169 (C). (**E** and **F**) Using different dyes, imaging at 48 HAI revealed two presumably dead host cells (DC) with increased fluorescence in close proximity of the HM in U6169 (E); no dead cells were revealed in Morocco (F). Staining of nuclei with propidium iodide (red) was indicative of cell death [insert in (E)]. SSV, fungal substomatal vesicle; MC, mesophyll cells; HM, haustorial mother cells.

Only one gene (MF474174) carried mutations in each *Pgt* mutant; 12 mutants had nonsense mutations, one mutant carried a splice-site disruptingmutation, and two mutants had the same nonsynonymous mutation producing valine to isoleucine (V128I) substitution (Fig. 2A and table S9). This *AvrSr35* candidate gene encoded a 578–amino acid protein with a predicted secretion signal peptide (fig. S3). The protein was larger than many previously identified effectors (*9*); it showed no similarity to proteins from other species within the protein databases, nor did it contain any detectable protein domains (fig. S4) (*7*). Gene expression analysis of a *Pgt*-infected susceptible wheat line showed increased amounts of the *AvrSr35* transcripts in the leaf tissues over the course of infection (fig. S3).

**Fig. 2:**
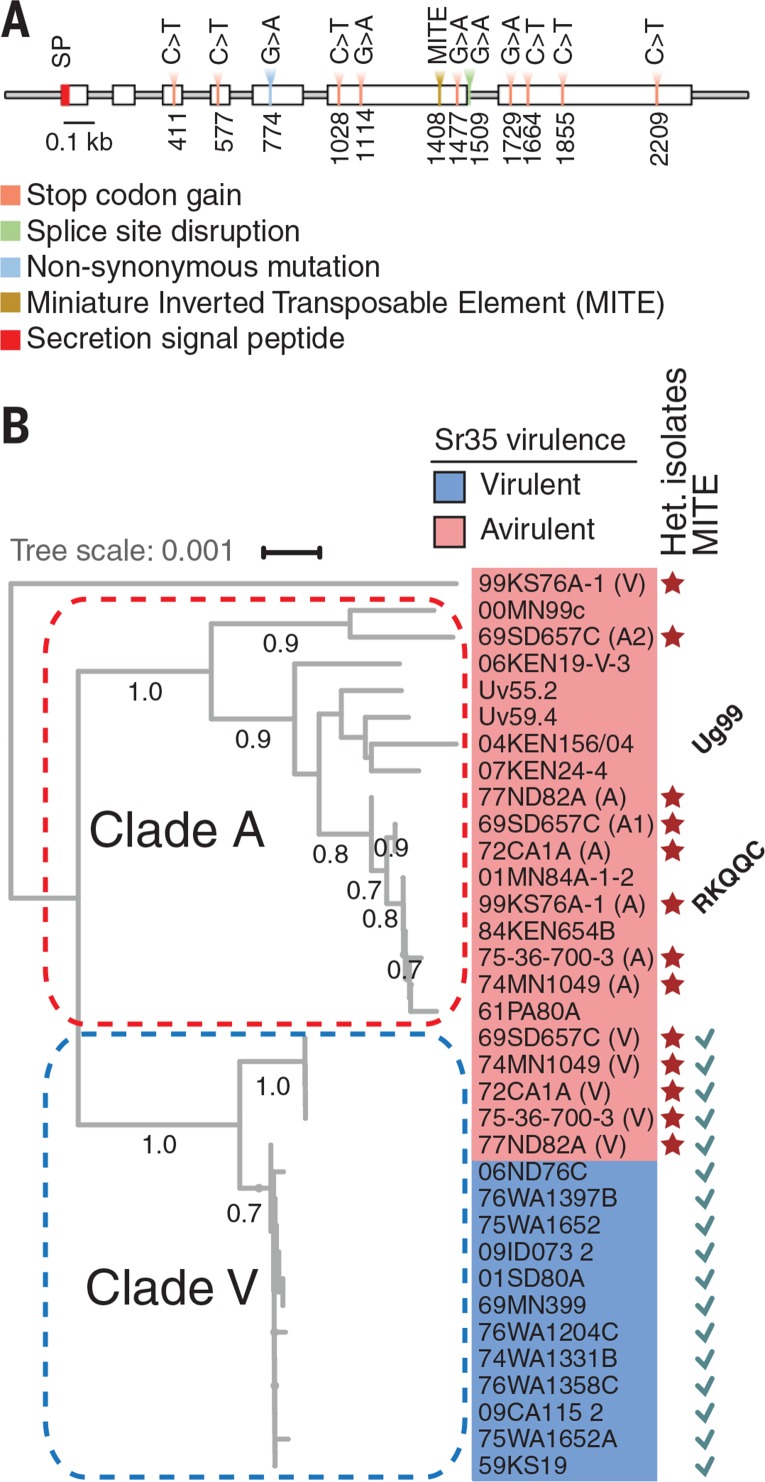
**Identification of the candidate *AvrSr35* gene. (A)** EMS-induced mutations and MITE insertion site in the *AvrSr35* gene. **(B)** Phylogeny of the *AvrSr35* gene in diverse Pgt isolates. The colored tree tips correspond to alleles originating from the *Sr35*-avirulent (red) and *Sr35*-virulent (blue) isolates. The sequences from the Pgt isolates heterozygous at the *AvrSr35* locus are marked with stars. The different *AvrSr35* alleles from these isolates have A or V appended to the sequence name. The sequences with MITE insertion are marked by checkmarks. The *AvrSr35* gene sequences form two major clades, A and V. Clade V includes the virulent allele with the MITE insertion. Bootstrap values above 0.7 are shown on the tree nodes. The second *AvrSr35* allele (accession number MF596174) from isolate 99KS76A-1 with a nonsense mutation in coding sequence was used as an outgroup.

To understand the origin of virulence to *Sr35* in the field, we resequenced *AvrSr35* from 12 *Sr35*- virulent and 15 *Sr35*-avirulent natural isolates (tables S10 and S11 and data S2). Phylogenetic analysis revealed twomajor clades (Fig. 2B). Clade A sequences had intact coding sequences and were found only in the *Sr35*-avirulent isolates, including 99KS76A-1 and Ug99, indicating that functional *AvrSr35* is required for triggering *Sr35* resistance against Ug99. Clade V sequences were preferentially found in the *Sr35*-virulent isolates, except for five *Sr35*-avirulent isolates (77ND82A, 72CA1A, 75-36-700-3, 69SD657C, and 74MN1049) that carry at least two *AvrSr35* gene copies fromboth clades (*7*). Clade V had the miniature inverted transposable element (MITE) in exon 6, resulting in the premature stop codon. Because even less severe *AvrSr35* truncations detected in the *Pgt* mutants (Fig. 2A) caused *Sr35*-avirulence function loss, this MITE insertion is predicted to produce a nonfunctional protein. These results suggest that transposon-mediated disruption of *AvrSr35* resulted in the origin of natural *Sr35-*virulent *Pgt* isolates. It is likely that the loss of *Avr* factors is facilitated by transposon proliferation in the rust genomes, which display the higher abundance of mobile elements compared with other fungi (*9*, *10*), contributing to the erosion of plant *R* genes conferring resistance to rusts.

The ability of the wheat *Sr35* gene to recognize the fungal *AvrSr35* and trigger HR was confirmed by coexpressing thewild-type andmutated *AvrSr35* and *Sr35* gene constructs (table S12) in *Nicotiana benthamiana* leaves (Fig. 3, fig. S5, andmovie S1). Even though the coexpressed wild-type constructs induced HR, the coexpression of truncated *AvrSr35* with wild-type *Sr35* failed to induce HR. No HR was evident in the combination of wild-type *AvrSr35* with either of the two loss-of-function *sr35* alleles, one with mutation (K206L) in the Ploop domain required forHR (*7*) andanotherwith three mutations found in the leucine-rich repeat (LRR) domain of the Ug99-susceptibleM1120mutant of diploid wheat accession G2919 (*6*). The expression of individual wild-type constructs also failed to elicit HR (fig. S6). Because loss-offunction mutations in either *AvrSr35* or *Sr35* resulted in both HR loss in *N. benthamiana* and a compatible interaction between *Pgt* and wheat, direct or indirect recognition of *AvrSr35* by *Sr35* appears to induce a resistance response in wheat. Consistent with this conclusion, the infiltration of the AvrSr35 protein caused HR in the leaves of a *Sr35*+ but not in the leaves of a *Sr35*– wheat lines (Fig. 3E).

**Fig. 3:**
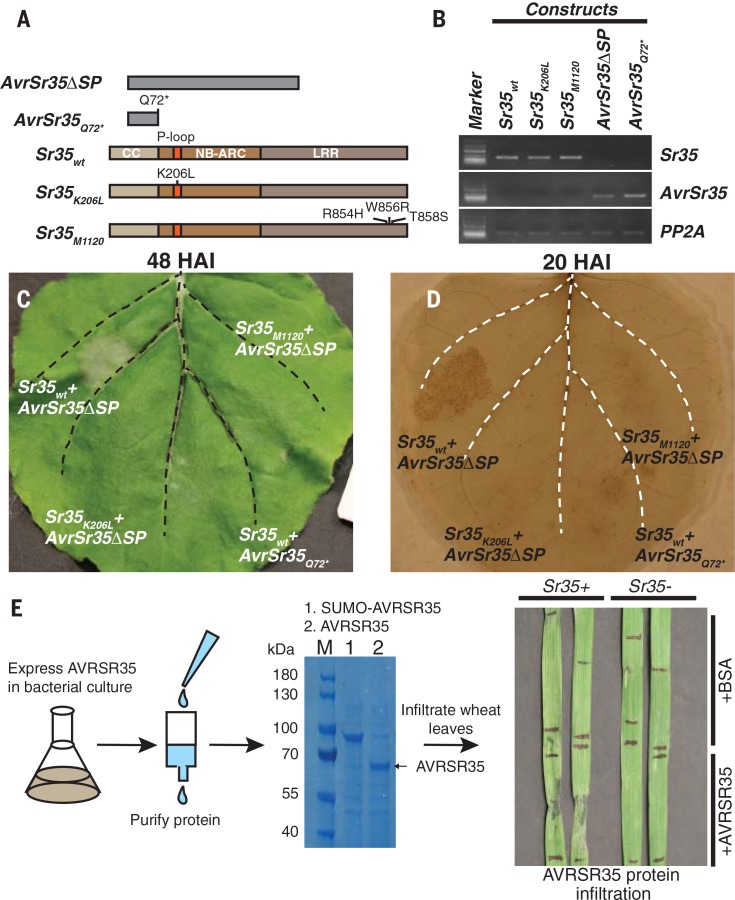
**AvrSr35 triggers Sr35-dependent cell death in *N. benthamiana* and wheat leaves. (A)** The *AvrSr35* and *Sr35* gene constructs were delivered into the *N. benthamiana* leaves by *Agrobacterium tumefaciens* infiltration. **(B)** Expression of gene constructs in *N. benthamiana* was validated by reverse transcription polymerase chain reaction (RT-PCR). The protein phosphatase 2A (PP2A) gene was used as an internal control (*7*). **(C)** Coinfiltration of *N. benthamiana* leaves with wild-type and mutant *Sr35* and *AvrSr35* constructs. The images were taken 48 to 72 HAI. **(D)** The accumulation of reactive oxygen species accompanying HR was assessed by staining leaves with 3,3′-diaminobenzidine 20 to 24 HAI. Each leaf was infiltrated at four sections formed by a midvein and two secondary veins (dashed lines). **(E)** Infiltration of the AvrSr35 protein into the leaves of *Sr35*+ and *Sr35*– wheat lines. The SDS–polyacrylamide gel electrophoresis analysis of AvrSr35 before (1) and after (2) small ubiquitin-related modifier (SUMO) protease treatment. Bovine serum albumin (BSA) was used as a negative control.

In *N. benthamiana* cells, fluorescently tagged Sr35 and AvrSr35 proteins, either coexpressed together or expressed individually, colocalized in the same subcellular compartment (Fig. 4, A to C). Colocalization of coexpressed Sr35:GFP protein fusion and the endoplasmic reticulum (ER) marker suggest that Sr35 and AvrSr35 expressed in *N. benthamiana* are likely associated with the ER (Figs. 4C and fig. S7). To investigate whether colocalized Sr35 and AvrSr35 interact in planta, we used bimolecular fluorescent complementation (BiFC) (Fig. 4D and figs. S8 and S9). The complementary AvrSr35 and Sr35 fusion proteins coexpressed in *N. benthamiana* produced a fluorescence signal that is consistent with a protein-protein interaction. The nonsynonymous mutations (*sr35*_M1120_ allele) affecting the LRR domain reduced the fluorescent signal intensity implicating the LRR domain in the Sr35- AvrSr35 interaction. These results suggest that the Ug99-susceptibility of wheat mutant M1120 (*6*) is associated with the inability of sr35_M1120_ to interact effectively with AvrSr35. The coimmunoprecipitation of epitope-tagged Sr35 and AvrSr35 expressed in *N. benthamiana* leaves supported the BiFC results, indicating that these proteins are capable of interacting in plant cells (fig. S10).

**Fig. 4:**
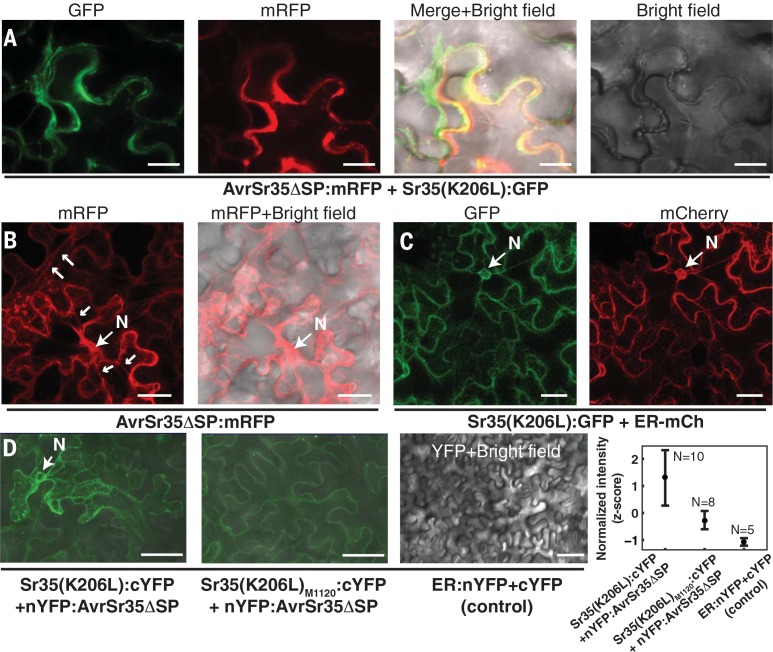
**Sr35 and AvrSr35 proteins colocalize in plant cells and interact. (A)** Coexpressed fluorescently tagged Sr35 and AvrSr35 colocalized in the *N. benthamiana* leaf epidermal cells. Scale bar, 20 µm. **(B)** In the *N. benthamiana* cells, the AvrSr35DΔSP:mRFP protein fusion accumulated in the ER strands (small arrows) and perinuclear space. Scale bar, 10 µm. **(C)** The Sr35(K206L):GFP protein fusion colocalized with the ER marker ER-mCherry in *N. benthamiana* cells (fig. S7). **(D)** Bimolecular fluorescent complementation showed interaction between Sr35 and AvrSr35 in the *N. benthamiana* cells. Compared with wild-type Sr35, the fluorescence intensity was significantly reduced in the cells expressing sr35_M1120_ (Tukey’s test adjusted *P* = 7.4 × 10^−4^) and negative control (Tukey’s test adjusted *P* = 8.7 × 10^−5^). Scale bar, 50 µm. N, nucleus.

The identification of *AvrSr35* and *AvrSr50* (*11*) provides valuable tools for molecular surveillance and early detection of virulent fungal pathogen races, which can inform the deployment of resistance genes to prevent epidemics. *AvrSr35* can also be used to confirm the expression of the functional Sr35 protein in the resistance gene cassettes, allowing for *Sr35* to be quickly pyramided alongside other *R* genes. As more corresponding *R*-*Avr* gene pairs are identified, this information can guide the selection of complementary *R* genes targeting multiple avirulence factors to increase the durability of the deployed resistance gene pyramids and reduce the probability of spontaneous virulent *Pgt* strain origin.

## SUPPLEMENTARY MATERIALS

www.sciencemag.org/content/358/6370/1604/suppl/DC1

Data S1 and S2

References (12–30)

21 August 2017; accepted 9 November 2017

10.1126/science.aao7294
