# Supplementary material for: Variation in the AvrSr35 gene determines Sr35 resistance against wheat stem rust race Ug99
Source: Science. 2017 Dec 22;358(6370):1604–6. doi: 10.1126/science.aao7294 (PMC6518949; doi:10.1126/science.aao7294)
Supplement: Supplementary file 1 [file Science-358-1604-s1.pdf]

www.sciencemag.org/content/358/6370/1604/suppl/DC1

Supplementary Materials for

Variation in the *AvrSr35* gene determines *Sr35* resistance against wheat stem rust race Ug99

Andres Salcedo,* William Rutter,* Shichen Wang, Alina Akhunova, Stephen Bolus, Shiaoman Chao, Nickolas Anderson, Monica Fernandez De Soto, Matthew Rouse, Les Szabo, Robert L. Bowden, Jorge Dubcovsky, Eduard Akhunov[1†]

*These authors contributed equally to this work.
†Corresponding author. Email: eakhunov@ksu.edu

Published 22 December 2017, *Science* **358**, 1604 (2017)
DOI: 10.1126/science.aao7294

This PDF file includes:

- Materials and Methods
- Figs. S1 to S10
- Tables S2 to S9, S11, and S12
- References

Other Supplementary Material for this manuscript includes the following:
(available at www.sciencemag.org/cgi/content/full/358/6370/1604/DC1)

- Tables S1 and S10
- Data S1 and S2
- Movie S1

Materials and Methods

<u>Confocal microscopy</u>

Plant material: Seedlings (12 days old) from wheat lines Morocco (*Sr35*-) and U6169 (*Sr35*+) KS05HW14*4//Mq(2) 5* G2919-k/Lakin were inoculated by spraying with a suspension of *Puccinia graminis* (*Pgt*) urediniospores in the isoparaffin solvent Soltrol® 170 (Chevron Phillips Chemical, TX). Seedlings were incubated in a dew chamber (20°C) in dark conditions for 12 hours followed by 1 hour incubation under light. Inoculated seedlings were kept in a growth chamber at 22°C with a 16 h / 8 h (day/night) photoperiod. Leaf samples were collected at 24, 48 and 72 hours after inoculation (HAI).

Staining: Infected leaves were fixed and cleared at room temperature in 95% ethanol for 5 days. Leaf tissues were washed twice by incubating for 15 min in 50% ethanol followed by 15 min incubation in 0.5M NaOH. Then samples were washed three times with water and kept for 30 min in 50 mM Tris-HCl buffer (pH 5.8). Staining of leaf samples was performed by incubating them for 5 min in 2 ml of 0.1% Uvitex 2B (Polysciences Inc.) followed by washing three times with ddH_2O. To stain the plant cell walls, samples were treated with 0.1% (w/v) acridine orange (Sigma) for 5 min. The excess of dye was removed by overnight incubation in 5 ml of 25% (v/v) glycerol. Fluorescence microscopy was carried out using a LSM 780 confocal laser scanning microscope (Zeiss) at 40X magnification. Uvitex 2B and acridine orange were detected by excitation at 405 nm and scanning with emission filters at 411-485 and 550-560 nm, respectively. Image data processing was carried out using Zeiss ZEN (Zeiss Efficient

Navigation) 2012 v8.1 and ImageJ software. At least 10 fungal infection sites in three biological replicates were analyzed for each stained leaf sample.

To detect dead cells in the leaf tissues, samples were stained for 15 min in a solution containing 20 ug/ml WGA-FITC dissolved in 50 mM Tris-HCl buffer (pH 7.5). Then samples were washed three times with 50 mM Tris-HCl buffer (pH 7.5), stained in 10 μg/ml water solution of propidium iodine (Life Technologies) followed by rinsing three times with ddH_2O. Fluorescence microscopy was carried out using a LSM 780 confocal laser scanning microscope (Zeiss) at 40X magnification. WGA-FITC and propidium iodine were detected by excitation at 488 and 561nm and scanning with filters at 493-584 and 584-718 nm, respectively (Fig. 1). At least 10 fungal infection sites in three biological replicates were analyzed for each stained leaf sample.

<u>The analysis of *Sr35*-based pre-haustorial resistance against stem rust</u>

Two genotypes of wheat with (U6169) and without (Morocco) the *Sr35* gene were inoculated with *Sr35*-avirulent *Pgt* race RKQQC. At least 10 fungal infection sites in three biological replicates were analyzed for each stained leaf sample. The leaf tissues were analyzed using a confocal microscopy 24 and 48 hours after inoculation (HAI). The staining with Uvitex 2B in combination with orange acridine was used to detect the fungal infection structures including substomatal vesicles (SSV), haustoria (H), haustorial mother cells (HM), and infection hyphae (IH) (Fig. 1). The staining with WGA-FITC and propidium iodine was used to identify the fungal tissues and the presence of dead cells in the leaf mesophyll of the infected plants (Fig. 1E and Fig. 1F). The collapse and increased fluorescence of individual plant mesophyll cells in close proximity to haustorial

mother cells (Fig. 1E), and the staining of nuclei in these mesophyll cells were considered as the evidence of cell death (*12*).

<u>EMS mutagenesis of *Pgt* spores</u>

The *Sr35*-avirulent *Pgt* isolate 99KS76A-1 (RKQQC race) was used to create *Sr35*-virulent mutants by treating with chemical mutagen ethyl methanesulfonate (EMS). The fresh *Pgt* urediniospores were collected from the leaves of susceptible cultivar Morocco 15 days after inoculation (DAI). A total of 120 mg of urediniospores were resuspended in 100 ml of the ethyl methanesulfonate (EMS) solution with 0.01% of Tween 20. Four different concentrations of EMS were used for mutagenesis: 0.1 M, 0.05 M, 0.01 M and 0.005 M. The urediniospore suspensions were agitated (80 rpm) for 2 hours in an orbital shaker at room temperature. Urediniospores mutagenized at different concentrations of EMS were pooled and collected by filtering through a polycarbonate membrane filter (Whatman, 0.22 µm) using a bottle-top vacuum filter system (Corning Inc.). The filter with urediniospores was washed by filtering 1L of ddH_2O and dried for 12-14 hours at room temperature. The spores were resuspended in isoparaffin oil (Soltrol 170) and used to inoculate one thousand 12-day old seedlings of *Triticum monococcum* accession G2919 (*Sr*35+) that was previously used for cloning the *Sr35* gene (*6*). Seedlings were incubated in a dew chamber at 20°C for 12 hours in dark followed by 1-hour incubation under light. Finally, seedlings were transferred to a growth chamber at 22°C with a 16-hour light and 8-hour dark photoperiod. The presence of pustules on the seedlings was evaluated 15 DAI. The spores were collected separately from 15 pustules, resuspended in Soltrol® 170 (Chevron Phillips Chemical, TX), and each suspension was used to re-

infect the seedlings of G2919 accession. This step was repeated twice for each mutant *Pgt* isolate. To ensure that plants inoculated by different mutagenized *Pgt* strains are not cross-infected by other rust spores, seedlings before transferring to growth chamber were covered by breathable cellophane bags. Each mutagenized strain of *Pgt* was multiplied by infecting susceptible wheat cultivar Morocco and validated for virulence to the *Sr35* gene by re-infecting the G2919 accession. Randomly selected set of mutants was pathotyped by infecting wheat lines that carry different known *Sr* genes (*13*) (Tables S1, S2).

To ensure that the obtained *Sr35*-virulent mutant strains were not escapes of other rust strains, and to validate that only *Sr35*-virulence was affected by EMS mutagenesis, five randomly selected mutants (M1, M7, M6, M9, M11) were analyzed by infecting a standard set of 20 differential wheat lines that carry different *Sr* genes (Table S1). This panel is broadly used for quick pathotyping of unknown *Pgt* isolates using the standard 5-letter code (*13*). In addition, the wheat cultivar Morocco (*Sr35*-) and G2919 accession of *T. monococcum* (*Sr35*+) were included as controls. For *Pgt* mutants M1 and M7, we assessed the virulence profiles on an expanded panel of wheat lines carrying different *Sr* genes (Table S2).

<u>The effect of *AvrSr35* gene mutations on *Pgt* virulence on the susceptible host</u>

The confocal microscopy of wheat leaves from compatible cultivar Fielder infected with the wild-type and mutant *Pgt* strains was performed to investigate the effect of the *AvrSr35* gene mutations on the pathogen's virulence. Four 2-week seedlings were infected with each of the four *Pgt* strains (wild-type, M1, M4 and M7). Leaf samples were collected three days after infection and stained using Uvitex 2B in combination with

orange acridine, as described above, and analyzed using the confocal microscope (Fig. S1).

The confocal microscopy analysis was also performed on the leaf samples collected 4 days after infection and stained for 15 min in a solution containing 20 ug/ml WGA-FITC dissolved in 50 mM Tris-HCl buffer (pH 7.5). For each detected fungal infection unit, we used ImageJ software to assess the area (in pixels) corresponding to the stained fungal structures. The average area was estimated for each plant based on the analysis of 3-5 infection units. The analysis of variance (ANOVA) was performed to test for differences in the fungal structure area among the leaf samples infected with the wild-type and mutant *Pgt* strains (Table S3).

We have also compared the growth of three EMS-mutants (M1, M4, and M7) and wild type *Pgt* strain by estimating the average sizes of uredia (pustules), the fungal structure producing spores, from images collected from the infected leaves of Fielder cultivar 15 days after infection. Four infected plants per *Pgt* strain were analyzed. Images were analyzed using the ASSESS (version 2) image analysis software for plant disease quantification from the American Phytopathology Society. The statistical significance of differences in the uredia sizes among the plants infected with the wild-type and mutant *Pgt* strains was tested using ANOVA (Table S3).

<u>RNA-seq analyses of susceptible wheat transcriptome infected with the wild type and mutant strains of *Pgt*</u>

To assess the effect of *AvrSr35* nonsense mutations on the *Pgt* interaction with the susceptible wheat host, we performed time-course analysis of leaf transcriptomes

obtained by infecting the susceptible wheat cultivar Fielder with either the wild-type *Pgt* isolate 99KS76A-1 or one of the three EMS mutants M1, M4 and M7 (Table S4). Total RNA was extracted from the infected leaf tissue at 24, 48, and 96 HAI as described previously (*8*). One microgram of high quality total RNA was taken for RNA sequencing (RNA-seq) library construction using the TruSeq RNA Sample Preparation kit v2 (Illumina). All RNA-seq libraries were prepared with the Biomek FXP Laboratory Automation Workstation (Beckman Coulter). The obtained RNA-seq libraries were analyzed with the 2100 Bioanalyzer (Agilent Technologies) and quantified with Qubit dsDNA BR Assay Kit (ThermoFisher Scientific). Indexed RNA-seq libraries were normalized to 10nM and then pooled in equal volumes, 6 libraries per pool. Each pool was sequenced in one lane of HiSeq 2500 (2x100 bp) at the University of Kansas Medical Center Genome Sequencing Facility. The obtained RNA-Seq data was used to annotate the expressed genes in the reference sequence of *Pgt* isolate 99KS76A-1, and also to assess the effect of mutations in the *AvrSr35* gene on the host's transcriptional response to infection. The RNA-Seq data is deposited to the NCBI database under BioProject PRJNA415853.

The quality trimming was performed according to the previously described protocols (*8*). The transcript abundance for the gene models from the wheat reference genome TGACv1 (*14*) was estimated from RNA-Seq data using *kallisto* (*15*) (NCBI GEO GSE106397). The analyses of differential gene expression were carried out using the R package *sleuth* that combines bootstrapping with linear modeling to estimate biological variance separately from inferential variance.

Differentially expressed genes were identified between the mock and wild-type *Pgt* inoculated wheat leaves collected at 24, 48 and 96 HAI, respectively (FDR $\leq$ 0.05) (Fig. S2). To characterize the wheat host biological pathways affected by interaction with the wild-type *Pgt*, we performed GO term enrichment analyses of differentially expressed genes and summarized results by clustering GO terms based on semantic similarity measures implemented in the REVIGO tool. This data was used to identify the wheat host genes involved in primary biotic defense stress responses during the compatible interaction with the pathogen (Fig. S2 and Table S5) (*8*, *16*, *17*, *3*).

To investigate whether the host transcriptional responses are affected by the nonsense mutations in the *AvrSr35* gene, we have compared the transcriptomes of leaves infected with the wild-type *Pgt* with the transcriptomes of leaves infected with the *Pgt* mutants (M1, M4 and M7) at the same time-points (Table S4). The two FDR levels, 0.01 and 0.05, have been used to identify the differentially expressed genes (Fig. S2).

<u>Sequencing the genome of *Pgt* isolate 99KS76A-1 (race RKQQC)</u>

To identify the *AvrSr35* effector we have mutagenized the American *Pgt* isolate 99KS76A-1 collected in 1999 in Kansas and classified as race RKQQC. Both RKQQC and Ug99 races of *Pgt* are avirulent to the *Sr35* gene (*6*). Before sequencing, the *Pgt* culture was purified by single-pustule isolation and pathotyped according to North American nomenclature system (*13*).

About 500 mg of freshly collected urediniospores were frozen in liquid nitrogen and ground using a mortar and pestle. Fungal DNA was isolated using the Omni Prep for Fungus DNA extraction kit (G-BioSciences, MO) according to the manufacturer's

protocol with the following modifications: The Molecular Grinding Resine ™ was not used and ground spores were directly re-suspended in 1 ml of the Genomic Lysis Buffer. 10 µl of RNAse A [10 mg/ml] (Qiagen, Hilden, Germany) and 10 µl Proteinase K solution (G-BioSciences, MO) were added to Genomic Lysis Buffer before incubation for 2 hours at 60°C. This method was used to sequence *Pgt* isolate 99KS76A-1 and the 15 *Pgt* mutants.

Genomic DNA from the wild-type *Pgt* was sequenced using three different sequencing platforms: Illumina MiSeq, Roche 454, and PacBio (Table S6, NCBI SRA database SRX1619626, SRX1619625, SRX1619623, SRX1619622). Standard library preparation protocols were followed for each platform.

For Illumina library construction, one microgram of high quality genomic DNA was fragmented with the Covaris S220 Focused – Ultrasonicator using the manufacturers recommended settings to obtain average DNA fragment length of 500bp. The NEBNext DNA Library Prep Master Mix was used for library preparation according to the NEB protocol. The obtained library was subjected to 500-900 bp size selection using the Pippin Prep™ system (Sage Science). The size-selected DNA library was analyzed using the 2100 Bioanalyzer (Agilent Technologies) and quantified with Qubit dsDNA HS Assay Kit (ThermoFisher Scientific). Sequencing runs were performed at the K-State Integrated Genomics Facility (IGF) on the MiSeq personal sequencing system (Illumina) using the 600 cycles MiSeq reagent v3 kit (Illumina) according to Illumina instructions. This method was used to sequence *Pgt* isolate 99KS76A-1 and the 15 *Pgt* mutants (Tables S6, S7).

The 454 sequencing library was prepared from 500 ng of genomic DNA with the GS FLX Titanium Rapid Library Preparation kit (Roche). The sample was sequenced with one 454 run using the Titanium chemistry at the K-State IGF followed standard Roche protocols (Table S6). The data is deposited to NCBI PRJNA313186.

The 3-10 kb PacBio libraries were constructed, quantified, and sequenced on one SMRT cell of PacBio RS II using P6C4 PacBio chemistry at UC Davis Genome Center (Table S6).

Illumina sequence data was processed using FASTX-Toolkit to remove adaptors, low quality bases (<20) and low-quality reads with less than 70% of bases having quality ≥20. Reads produced by 454 sequencing were adaptor- and quality-trimmed using the program Lucy with the default settings (sourceforge.net/projects/lucy).

Illumina paired-end reads generated for the *Pgt* isolate 99KS76A-1 were assembled using DISCOVAR *de novo* with the default parameters. To extend contigs, data generated using the Roche 454 and PacBio platforms were added using program SSPACE with “Minimum alignment length” = 100 and “Minimum identity of the alignment = 70”. Contigs longer than 1,000 bp have been retained. The contaminating sequence assemblies were excluded by performing BLASTN search in the NCBI’s non-redundant sequence database and retaining only contigs with the best hits to fungal sequences (e-value less than $1e^{-10}$ and alignment length more than or equal to 100 bp). The genome assembly and annotation are available from the NCBI PRJNA313186 and project website http://wheatgenomics.plantpath.ksu.edu/rustgenomics/.

Annotation of *Pgt* genome assembly

For predicting genes in the *Pgt* genome, we performed *de novo* assembly of RNA-Seq data (BioProject PRJNA415853) generated for total RNA isolated from the *Pgt*-infected leaf tissues (described in the next section). For RNA extraction, twelve day-old seedlings of susceptible wheat cultivar 'Fielder' were inoculated with the urediniospores of *Pgt* isolate 99KS76A-1, as previously described (*6*). Infected leaf tissues were collected at 24, 48, and 96 HAI and used for RNA-seq analysis.

Illumina 2 x 100 bp paired-end reads were quality filtered and assembled using program Trinity (*18*) with the following parameters: "--genome_guided_max_intron 50000 --SS_lib_type FR –jaccard_clip". The assembled contigs were used for the *Pgt* genome annotation. In addition, for the *Pgt* genome annotation, we have used 1) 15,979 gene models reported for the genome of *Pgt* strain CDL 75–36-700–3 (*9*), and 2) 22,321 transcripts reported for the Australian *Pgt* isolates. Gene models were predicted by mapping the transcripts to the *Pgt* genome assembly using the PASA pipeline (*19*). The coding regions of genes were predicted using the TransDecoder tool of the PASA pipeline (NCBI database PRJNA313186 and project website http://wheatgenomics.plantpath.ksu.edu/rustgenomics).

To estimate the completeness of genome assembly, the BUSCO program was utilized (*20*). BUSCO uses a set of 1,441 phylogenetically conserved genes to assess the proportions of completely and partially sequenced genes in a genome or transcriptome.

Re-sequencing 15 *Pgt* mutant strains and mutation discovery

The BWA-MEM program with the default parameters was used to align reads generated for 15 mutants and the wild-type strain to the reference genome assembly of *Pgt* isolate 99KS76A-1 (NCBI PRJNA313186) (Table S7).

Before variant calling, the reads in BAM files were locally re-aligned using the GATK (*21*) followed by the marking and removal of duplicated reads. The variant calling was performed using the GATK's UnifiedGenotyper (*21*) with the default settings. The raw variant calls were filtered to remove sites that had more than 2 allelic states or were monomorphic among the 15 mutant strains. Because EMS preferentially alkylates G residues, inducing primarily C-to-T and G-to-A transitions (*22*), only CG > TA mutations were selected. Additional filtering has been applied to read coverage data extracted for both wild-type and mutated allelic variants. Alleles were called only if they had coverage of at least two reads. The minimum coverage of 5 reads was used to call mutant alleles. To reduce the erroneous mutant calling due to the misalignment of duplicated paralogs or cross-contamination among the mutant *Pgt* strains during their isolation, the mutant sites that were detected in more than 3 different *Pgt* mutant strains have been excluded. In addition, the Fisher's exact test was applied to compare the depth of read coverage of mutant and wild-type alleles at each site in the wild-type and mutant strains. The sites showing the statistically significant difference at P-value $\leq 10^{-4}$ were retained (Dataset S1, Table S8). All mutations in the top candidate gene were confirmed by Sanger sequencing.

Identification and characterization of the *AvrSr35* gene candidate

We searched for candidate genes that had multiple nonsense or other strong effect mutations in the coding sequences across all 15 *Pgt* mutants (Dataset S1). The mutations detected in a single top candidate gene (NCBI accession number MF474174) were validated by the Sanger sequencing of amplicons generated for each of the 15 fungal mutants (Fig. 2A, Table S9). The pair of primers (Table S10) was designed to amplify the entire *AvrSr35* gene coding region.

The *AvrSr35* protein sequence (578 amino acids) was analyzed for the presence of a signal peptide cleavage site (Figs. 2A and S3). AvrSr35 was scanned for the presence of matches in the InterPro protein signature databases using InterProScan. AvrSr35 sequence matches in the NCBI protein database were searched using BLASTX. The size of the *AvrSr35* protein was compared with the sizes of the previously characterized effector proteins from flax (*23*) and poplar rust (*9*).

Structural modeling of the AvrSr35 protein

To overcome the lack of the AvrSr35 homologs in the protein databases, we predicted the secondary structure of AvrSr35 (Fig. S4) and compared it to a database of known protein 3D structures using the online I-TASSER server (*24*). I-TASSER uses a threading approach to match proteins to known 3D protein structures in The Protein Data Base (PDB), which as of 2017 contained over 138,000 deposited protein structures.

Diversity analyses of *Sr35*-virulent and *Sr35*–avirulent field strains of *Pgt*

To confirm the results of EMS mutagenesis, PCR amplicons for the *AvrSr35* candidate gene were generated and sequenced from a diverse set of *Pgt* field isolates. These isolates were collected at different locations and years and validated as *Sr35*-virulent (12 isolates) and *Sr35*-avirulent (15 isolates) (Table S11, Dataset S2).

The *AvrSr35* candidate gene region including entire coding sequence was amplified from each isolate (see the list of primers in Table S10) and sequenced from both strands using Sanger approach with multiple sequencing primers spanning the entire gene. The Sanger reads generated using the ABI3730xl (Applied Biosystems) system were processed and assembled into contiguous sequences using Sequencher 4.8 (Gene Codes). If PCR produced a single-band product on the agarose gel, it was sequenced directly. If isolate produced two or more (due to the presence of an additional paralogous gene copy in one of the two nuclei) PCR bands on the agarose gel, the PCR products were TA-cloned into the pGEM®-T Easy vector followed by Sanger sequencing of four to eight independent clones.

Sequences were aligned using program MUSCLE with the default settings. The phylogenetic tree was constructed using the Neighbor-Joining method applying the maximum composite likelihood substitution model implemented in program MEGA 6.06. The phylogenetic tree testing was performed utilizing the bootstrap method with 1,000 replicates. The sequence of highly divergent non-functional second allele (due to 1 bp deletion at 1,320 bp of CDS; NCBI accession number MF596174) of the *AvrSr35* gene identified in isolate 99KS76A-1 was used to root the tree.

Infiltration of resistant (*Sr35+*) and susceptible (*Sr35-*) wheat lines with the AvrSr35 protein

The coding region of the *AvrSr35* gene candidate without the deduced signal peptide (SP) was amplified from the pGEMT-cDNA-AvrSr35 (Table S10). The PCR fragment was cloned into the expression vector pET SUMO vector (Champion^{TM} pET SUMO Protein Expression System kit, Thermo Fisher Scientific) according to the manufacturer's instructions. The pET SUMO vector includes coding sequence for a small ubiquitin-like modifier (SUMO) that when N-terminal fused with the cloned coding sequence allows for effective expression and purification of proteins in *E. coli*. The recombinant plasmid was transformed into *E. coli* BL21 (DE3) chemically competent cells (Thermo Fisher Scientific). The SUMO-fused construct was validated by Sanger sequencing. The protein synthesis was induced by adding isopropyl-b-D thiogalactopyranoside (IPTG).

A single colony of *E. coli* BL21 (DE3) with the pET-SUMO-AvrSr35-ΔSP construct (Table S10) was grown overnight in 5 ml of LB media with 1% glucose and 50 µg/ml of kanamycin. The half of this overnight culture was transferred into 500 ml of LB media (1% of glucose, 50 µg/ml of kanamycin) and incubated at 37°C on rotary shaker (250 rpm) until cell density reached OD_{600} = 0.5. The protein synthesis was induced by adding isopropyl-b-D thiogalactopyranoside (IPTG) (Thermo Fisher Scientific) to a final concentration of 0.5 mM. The cell culture was then incubated overnight at 25°C in a rotary shaker (250 rpm).

Cells were harvested by centrifugation at 10,000 x g for 15 min at 4°C. Cell pellet was suspended in 50 ml of lysis buffer (50mM sodium phosphate buffer pH 6.8, 300 mM NaCl, 10mM imidazole (Thermo Fisher Scientific), 10 mM phenylmethylsulfonyl

fluoride (Thermo Fisher Scientific) and 0.05 mg of lyzosyme (Thermo Fisher Scientific). The suspension was incubated for two hours in a cold room (~4 – 6°C) and then was subject to three cycles of freezing and thawing using liquid nitrogen and a water bath set at 42°C. Cell lysate was harvested by centrifugation at 10,000 x g for 20 min at 4°C. The upper phase was transferred to a 50 ml tube and kept on ice.

The recombinant protein was purified using the HisPur Ni-NTA spin columns (Thermo Fisher Scientific). Columns were equilibrated using two resin-bed volumes of equilibration buffer (50mM sodium phosphate buffer pH 6.8, 300 mM NaCl, 10 mM imidazole) followed by centrifugation at 700 x g for 2 min at 4°C. The Ni-NTA resin was washed with 8 resin-bed volumes of wash buffer (50mM sodium phosphate buffer pH 6.8, 300 mM NaCl, 20 mM imidazole) followed by centrifugation at 700 x g for 2 min at 4°C. The recombinant protein was collected from the column by five successive elutions, using a bed volume of elution buffer (50 mM sodium phosphate buffer pH 6.8, 300 mM NaCl, 150 mM imidazole) followed by centrifugation at 700 x g for 2 min at 4°C. Total protein was quantified using Bradford method and analyzed in a 10% SDS-PAGE.

To obtain native protein, SUMO fusion was cleaved off by incubating 5 mg of purified recombinant protein with 100 Units of SUMO protease (Thermo Fisher Scientific) in 1X SUMO protease buffer for 16 hours at 4°C. Cleaved protein was analyzed in a Coomassie stained 10% SDS-PAGE to confirm the N-terminal cleavage (Fig. 3E). Cleaved protein was dialyzed using a Slide-A-Lyzer ™ Dialysis kit (Thermo Fisher Scientific). The Slide-A-Lyzer cassette containing the cleaved protein was subjected to overnight dialysis in a cold room with stirring in 5 L of ddH_20. After dialysis, the protein was purified using the HisPur Ni-NTA spin columns (Thermo Fisher

Scientific). Columns were equilibrated with two resin-bed volumes of ddH_20, followed by centrifugation at 700 x g for 2 min at 4°C. Dyalized protein was passed through the column by centrifugation at 700 x g for 2 min at 4°C. Then, the flow-through fraction containing the AvrSr35 protein was frozen in liquid nitrogen and lyophilized for at least 24 hours in Labconco FreeZone® Benchtop Freeze Dry Systems (Labconco Corporation) at a pressure vacuum of 0.08 mBar and a condenser temperature of -50 °C. The lyophilized protein was dissolved in 1 ml of cold 1X PBS (Phosphate Buffer Saline). The protein concentration was determined by the Bradford method.

For the protein infiltration, we used 12 days-old seedlings of hexaploid wheat cultivar Marquis without of the *Sr35* resistance gene (*Sr35*-) and line Marquis*5/G2919 with the introgression of resistance gene *Sr35* (*Sr35*+) (Fig. 3E). The AvrSr35 protein was diluted to 0.1 mg/ml in 1X PBS and infiltrated into the abaxial side of the first leaf using a 0.5 ml syringe. The Bovine Serum Albumin (BSA) dissolved in 1X PBS at 0.1 mg/ml was infiltrated into the same leaf to test if the hypersensitive reaction (HR) can be caused by a non-specific protein. Infiltrated seedlings were then transferred to growth chamber and maintained at 22°C with photoperiod of 16 hours. HR was evaluated 24 hours after infiltration (Fig. 3E).

Infiltration of *N. benthamiana* leaves with *Agrobacterium*

The full-length coding sequence of the candidate *AvrSr35* gene was obtained by RT-PCR of total RNA isolated from the 2-week old wheat seedlings infected with the RKQQC race of *Pgt*. The entire coding region of *AvrSr35* was amplified from the cDNA obtained after reverse transcription has been amplified using gene-specific primers (Table

S10) amplifying the entire coding region. The PCR product was subcloned into the pGEM5 vector and both strands of 5 independent plasmid clones confirmed to be identical using a Sanger sequencing approach. The clone was used for creating all of the derivatives of the *AvrSr35* gene constructs.

The gene fragments were amplified using the Gateway-compatible *attB*-modified primers and cloned into the pDONR ™/Zeo vector (Thermo Fisher Scientific). The constructs were validated by Sanger sequencing. The resulting entry clone was used to transfer segments of a gene into Gateway-compatible the pIPKd004 binary vector. The pAvrSr35-ΔSP-TDNA construct without the 26 amino acids-long signal peptide was created by subcloning the amplified fragment of the gene. The construct pAvrSr35(Q72*)-ΔSP-TDNA with the truncated version of the *AvrSr35* corresponding to the mutated gene variant in the M11 *Pgt* mutant (Table S9, S12) was created by amplifying the coding region between positions 76-214 bp. All constructs were validated by Sanger sequencing.

Three *Sr35* gene constructs were also created in the pIPKb004 vector, one with the full-length ORF and the two with the non-functional mutated variants of the *Sr35* gene (Table S12). The construct pSr35(K206L)-TDNA harbored an amino acid change K206L in the P-loop of the NB-ARC domain that was shown to be critical for nucleotide binding and triggering HR in other NBS-LRR proteins (*25*). The pSr35v1120-TDNA construct included the loss-of-function mutant variant of the *Sr35* gene, $sr35_{1120}$ identified in the EMS mutagenized Ug99-susceptible line *T. monoccoccum* G2919 (mutant family M1120) that was described previously (*6*). The $sr35_{1120}$ harbors three amino acid changes

(R854H, W856R, and T858S) in the LRR domain of the *Sr35* gene rendering it ineffective against the Ug99 and RKQQC races of *Pgt* (*6*).

The pIPK004 binary constructs were transformed into the *Agrobacterium tumefaciens* strain C58C1. For co-infiltrations, bacterial cultures containing each binary construct were grown to OD_{600} = 0.6 and mixed in equal proportions immediately prior to infiltrations. All *N. benthamiana* infiltrations were performed on 4-5week old plants. Each *N. benthamiana* leaf was infiltrated at four separate sections formed by mid-vein and two secondary veins (shown by black and white dashed lines on Fig. 3). The size of infiltration spot within each section was about 4 cm^2.

Leaves were scored between 48 and 72 hours post infiltrations at the first visible signs of a hypersensitive response. Co-infiltrated leaves were assayed for the presence of reactive oxygen species. For this purpose, leaves were cut 20-24 hours after infiltration, before visible signs of a HR, stained for 9 hours in a 3’3-DAB (1mg/mL, PH 3.8) solution, and subsequently cleared by boiling in 96% ethanol.

The *in planta* expression of each construct was assayed by extracting total RNA from leaf sections infiltrated with individual agrobacterium cultures using Trizol reagent, following the manufacturers protocols (Life Technologies). RNA samples were treated with DNase and used to generate cDNA, which were tested using gene specific primers for *Sr35*, *avrSr35*, and the house keeping gene NbPP2A (TC21939).

<u>Testing the ability of the tagged *Sr35* and *AvrSr35* genes to induce HR in *N. benthamiana*</u>

To ensure that the fusion of the *Sr35* and *AvrSr35* genes with the epitope tags (FLAG or HA) or fluorescent proteins (mRFP, GFP or nYFP) does not affect their

activity, we have tested the ability of the tagged proteins to trigger HR in *N. benthamiana* leaves (Fig. S5). The visible HR symptoms in the combinations of the AvrSr35 and Sr35 proteins fused with different epitope or fluorescent tags were used as the evidence of protein functionality.

Sub-cellular localization in *Nicotiana benthamiana* leaf epidermis cells

Florescent fusion constructs were produced by cloning the coding sequence of *AvrSr35* (AA27-578) and *Sr35* into the binary pSITE-4NA (monomeric red fluorescent protein fusion) and pSITE-2NB (enhanced green fluorescent protein fusion) Gateway destination vectors, respectively. Constructs were confirmed using Sanger sequencing, transformed into *Agrobacterium* strain C58C1, and infiltrated into the *N. benthamiana* leaves as described above. To increase confocal image resolution and depth, leaf sections were cut and soaked in perfluorodecalin (PFD). Plasmolysis was induced by adding a 0.8M mannitol solution. Leaf sections were imaged using a Zeiss 780 confocal microscope. The mCherry-labeled endoplasmic reticulum (ER) marker was the signal peptide of *Arabidopsis* wall-associated kinase 2 (AtWAK2) with the ER retention signal His-Asp-Glu at the C terminus (*26*). Plasma membrane marker was GFP-labeled RIN4 protein (*27*).

Co-immunoprecipitation assay

Epitope tags Human influenza hemagglutinin (HA) and DYKDDDDK (FLAG-tag) were added to the *AvrSr35* and *Sr35* genes, respectively, by PCR with the modified primers carrying epitope encoding sequences. PCR products were cloned into the binary

Gateway destination vector pIPKb004 to produce the plasmids pSr35-TDNA-n-Flag with the FLAG epitope on N-terminal end of *Sr35* and the plasmid pAvrSr35-ΔSP-TDNA-c-HA with the HA epitope on the C-terminal end of *AvrSr35*. Individual constructs were transformed into *Agrobacterium tumefaciens* strain C5851. The *Agrobacterium* strain cultures carrying binary constructs were grown to $OD_{600} = 1$ and mixed in equal proportions immediately prior to infiltrations. All *N. benthamiana* infiltrations were performed on 4- to 5-week old plants; the controls used were non-infiltrated *N. benthamiana* and single infiltration with each construct. Protein complex co-immunoprecipitations were made using μMACS anti-HA and anti-DYKDDDDK (FLAG-tag) isolation kits (Miltenyi Biotec). Both column bound and un-bound protein fractions were separated in the 10% SDS-PAGE until the dye front reached the bottom of the gel. The gel-separated proteins were transferred to the polyvinylidene difluoride (PVDF) membranes for 1 hour at 100 V, and rinsed once with the TBS buffer (10 mM Tris-HCl pH 8.0, 150 mM NaCl). The membranes were blocked using 5% BSA (Sigma) in the TBST solution (10 mM Tris-HCl pH 8.0, 150 mM NaCl, 0.1% Tween 20) by incubating overnight at 4°C with gentle shaking. Then the membranes were incubated for 1 h at RT with anti-HA-HRP (dilution 1:6,000) (Miltenyi Biotec) in 5% BSA dissolved in the TBST followed by 4 washes (10 min each) using TBST. Proteins were detected using Super Signal West Femto Chemiluminescent Substrate (Thermo Scientific Inc.) following the manufacturer's instructions. The images were acquired using the Odyssey Fc Imaging System (LI-COR Biosciences) and analyzed using Image Studio™ Lite v 3.1 (LI-COR Biosciences) (Fig. S10).

Bi-molecular fluorescent complementation (BiFC) assay

Interaction between the *AvrSr35* and *Sr35* genes was tested *in planta* by agro-infiltration of complementary fusion constructs with either the N–terminal domain (1–174 AA) or the C–terminal domain (175–239 AA) of the yellow fluorescent protein (nYFP or cYFP, respectively) into the *N. benthamiana* leaves. Interaction between the gene constructs was inferred based on the appearance of YFP-specific fluorescence signal. As a negative control, ER-targeted nYFP along with the cYFP construct were co-expressed in *N. benthamiana* resulting in no fluorescent signal (Fig. 4D).

To preclude the triggering of HR, the *Sr35* gene constructs used for infiltration carried the K206L mutation in the P-loop of the NB-ARC domain (Sr35:p. K206L) (Table S12). This mutation has been shown to inhibit nucleotide hydrolyses and downstream signaling in other NBS-LRR resistance proteins, but should not interfere with target binding. Constructs with the *Sr35* gene variant found in the susceptible EMS-mutagenized *T. monococcum* line M1120 (R854H+W856R+T858S) and ER-nYFP+cYFP were used as negative controls (Fig. 4D). The *Sr35* gene variants were created as C-terminal fusions with the cYFP (Table S12). The wild-type *AvrSr35* gene without the signal peptide (27-578 AA) was an N-terminal fusion with nYFP (Table S12). The level of construct expression in *N. benthamiana* leaves was tested by RT-PCR as described above. Also, to ensure that the decrease in fluorescence observed with the mutant *Sr35*$_{M1120}$ constructs is not due to reduction in protein stability, we tested the expression of these proteins by immunoblotting using the anti-GFP showing affinity to cYFP (Fig. S9).

We have quantified the intensity of fluorescence using images obtained for at least five independently transformed *N. benthamiana* plants for each construct combination. For each plant, we have analyzed from two to three leaves. The image analysis was performed using the ImageJ software by estimating the maximum fluorescence intensity in at least 30 non-overlapping windows covering entire cell on the confocal microscope image. To avoid the impact of variation across experimental measurements performed at different time or on different plants, the estimates of maximum fluorescence intensity within each experiment were normalized by calculating z-score (standard score). The z-score was calculated by subtracting from each measurement the mean value of the dataset and dividing it by the standard deviation of the dataset. The normalized z-score values were averaged for each plant and used to compare the level of fluorescence among the *N. benthamiana* plants agro-infiltrated with different constructs (Fig. 4D). The analysis of variance (ANOVA) was used for assessing the effect of different construct combinations on the level of fluorescence followed by the 'post-hoc' Tukey's test to assess the significance of individual means (Fig. 4D).

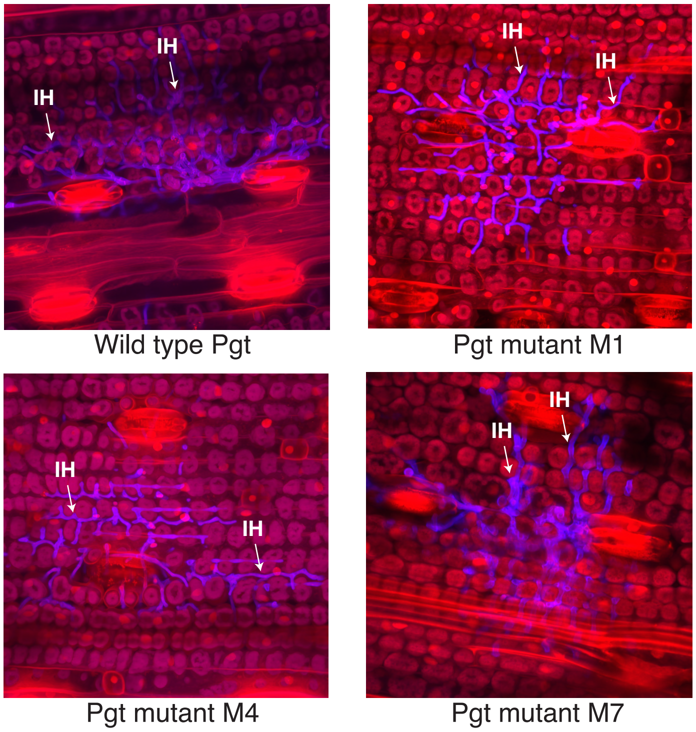

Fig. S1

Comparative analysis of fungal infection hyphae (IH) development in the wild-type and mutant *Pgt* strains. The leaves of susceptible cultivar Fielder (*Sr35*-) collected 72 hours after infection (HAI) were stained with Uvitex 2B and orange acridine. Fungal infection hyphae are stained blue. The initial stages of fungal hyphae development between the wild-type and three mutant *Pgt* strains did not show substantial differences.

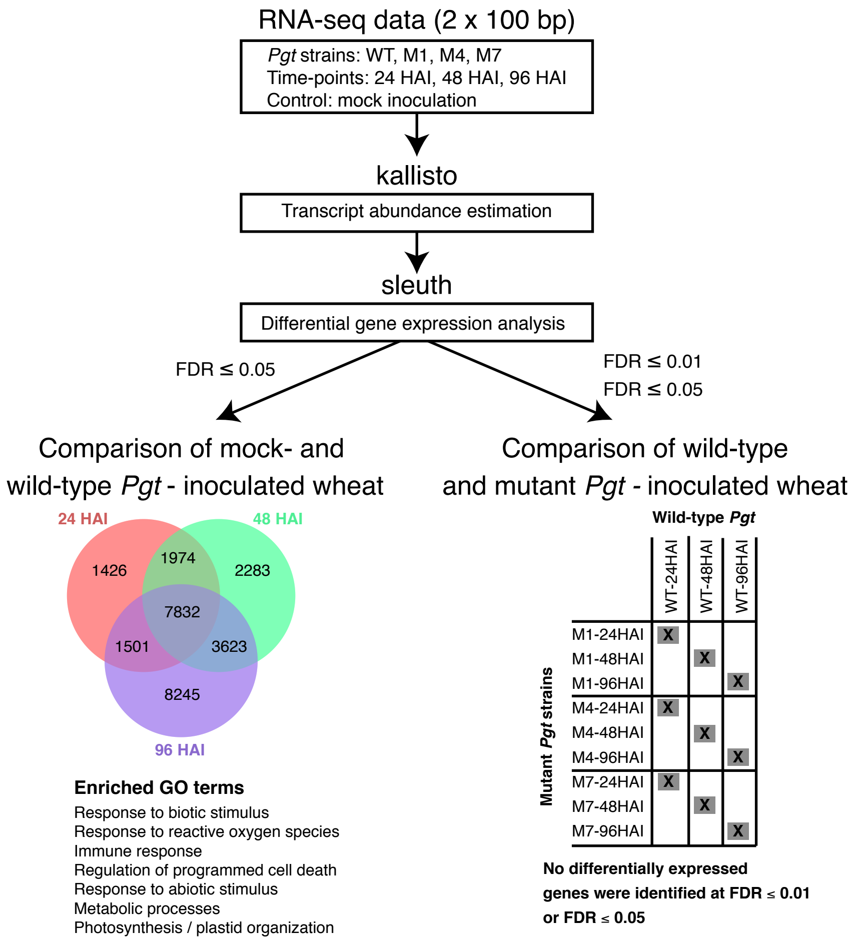

Fig. S2

RNA-Seq analysis of the leaves of susceptible cultivar Fielder inoculated with the wild-type *Pgt* isolate 99KS76A-1 and *Pgt* mutants M1, M4 and M7 (NCBI GEO GSE106397). *Pgt*-inoculated leaves were collected at 24, 48 and 96 HAI. The differentially expressed genes identified between the mock and wild-type *Pgt* inoculated wheat leaves were enriched in the GO terms previously known to be implicated in primary defense responses. At the two FDR levels, 0.01 and 0.05, we failed to detect any wheat genes that were differentially expressed between the leaves infected with the wild-type *Pgt* and its derived mutants.

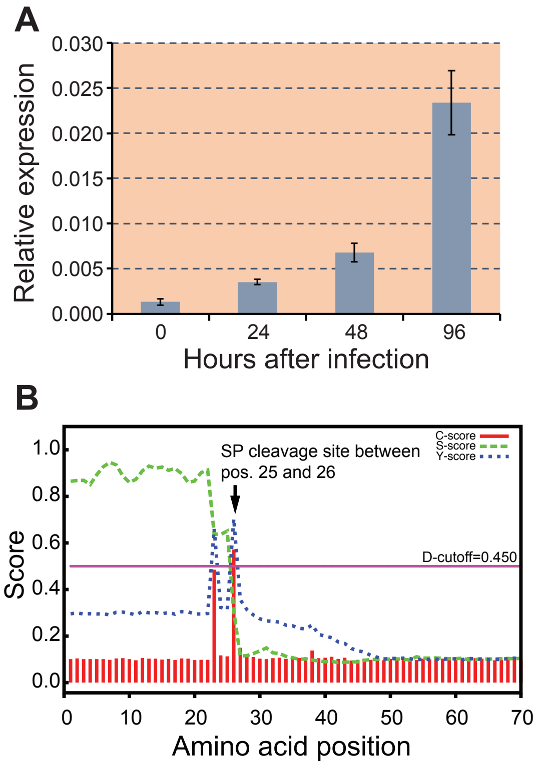

Fig. S3

(A) qRT-PCR determination of the *AvrSr35* transcript levels relative to tubulin in the infected leaf tissues of susceptible wheat cultivar Morocco. RNA was isolated from samples collected 0, 24, 48 and 96 hours after inoculation. The transcript levels are expressed using the $2^{-\Delta Ct}$ method. Error bars correspond to the SEM based on six biological and two technical replicates. (**B)** Prediction of secretion signal peptide (SP) using SignalP v.4.1 software (*28*). The first 70 N-terminal amino acids of the AvrSr35 protein are shown. The SP cleavage site was detected between amino acids 25 and 26. The output of SignalP shows the values of raw cleavage site score (C-score), signal

peptide score (S-score) and combined cleavage site score (Y-score). The D-cutoff, a weighted average of the mean S-score and the maximum Y-score, is used to identify SPs.

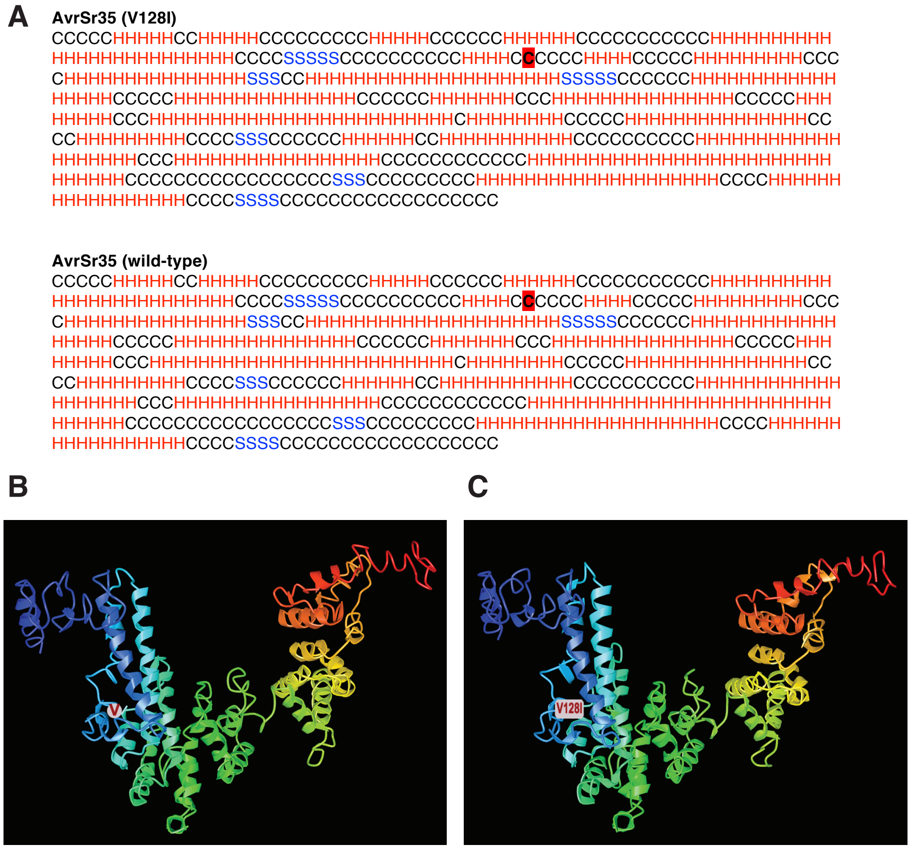

Fig. S4

The secondary and tertiary structure modeling of the AvrSr35 protein using I-TASSER. In the absence of the secretion signal peptide I-TASSER produced a secondary structure prediction of 25 helices and 6 strands interspersed with coil domains. The top predicted tertiary structure for wild-type AvrSr35 achieved a predicted template modeling score (TM-SCORE) of 0.37±0.13 and a predicted Root-Mean-Square Deviation (RMSD) score of 15.1±3.5Å. The top predicted tertiary structure for V128I mutant protein was similar (TM-SCORE = 0.38±0.13; RMSD = 14.9±3.6Å). In the experimentally-derived 3D protein structures within the PDB, the top 5 most similar structures were the components of nuclear pore complexes from different species. The V128I substitution (highlighted in red) found in the AvrSr35 protein in *Pgt* mutants M1 and M6 did not have substantial

effect on the predicted secondary (**A**) and tertiary (**B, C**) structure of the AvrSr35 protein. H – Helix, S – Strand, and C – Coil.

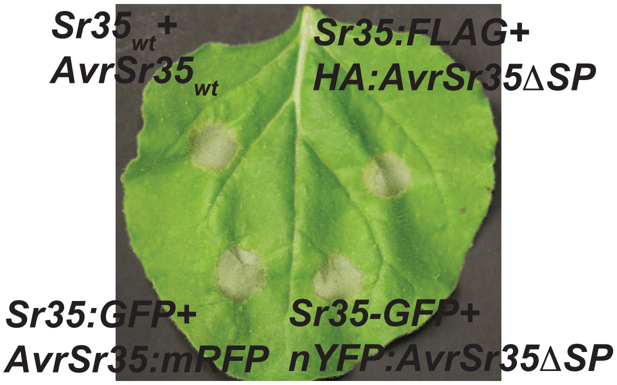

Fig. S5

Agro-infiltration of *N. benthamiana* leaves with the constructs expressing the *AvrSr35* and *Sr35* genes tagged with the HA / FLAG epitopes or fluorescent proteins showed HR symptoms. The *Agrobacterium* cultures transformed with the *AvrSr35* and *Sr35* gene-containing constructs were grown to OD_{600} = 0.6. Co-infiltration was performed with cultures mixed in 1:1 ratio. The images were taken 48-72 HAI at the first visible signs of HR.

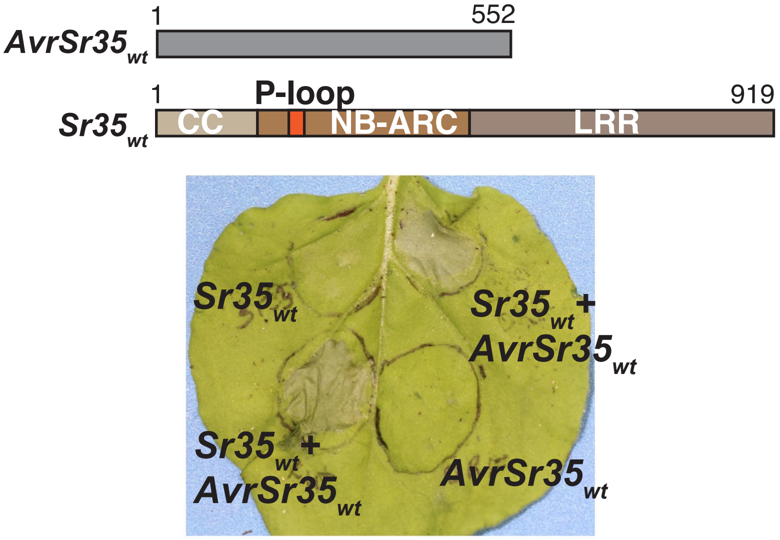

Fig. S6

Agro-infiltration of *N. benthamiana* leaves with the constructs expressing wild-type variants of the *AvrSr35* (without secretion peptide) and *Sr35* genes. The length of synthesized proteins (in the number of amino acids) is shown above the depicted constructs. The *Agrobacterium* cultures transformed with the *AvrSr35* and *Sr35* gene constructs were grown to $OD_{600} = 0.6$. Co-infiltration was performed with cultures mixed in 1:1 ratio. The size of infiltration spot within each leaf section was about 4 cm^2. The images were taken 48-72 HAI at the first visible signs of HR.

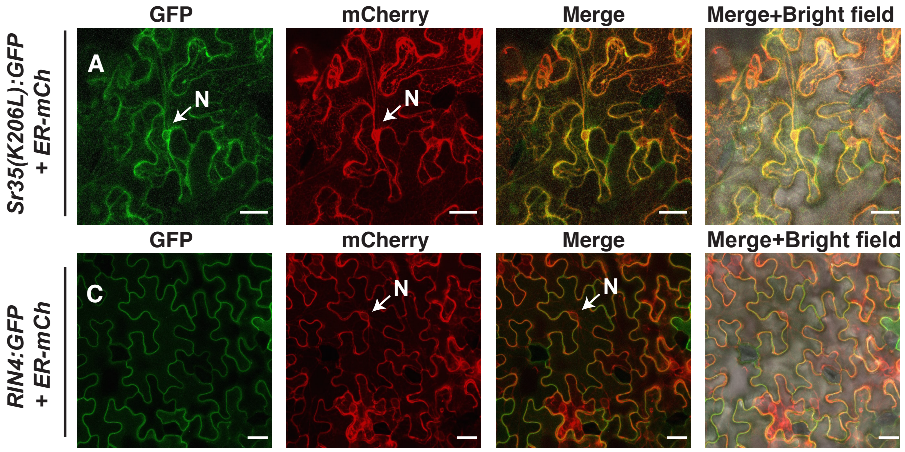

Fig. S7

A. When expressed in *N. benthamiana*, the fluorescence of the Sr35(K206L):GFP and endoplasmic reticulum (ER) marker ER-mCherry co-localized in the ER. **B.** In *N. benthamiana*, the ER-mCherry marker showed a distribution different from that of the RIN4:GFP fluorescent protein associated with the plasma membrane (PM). Scale bar: 20 μm.

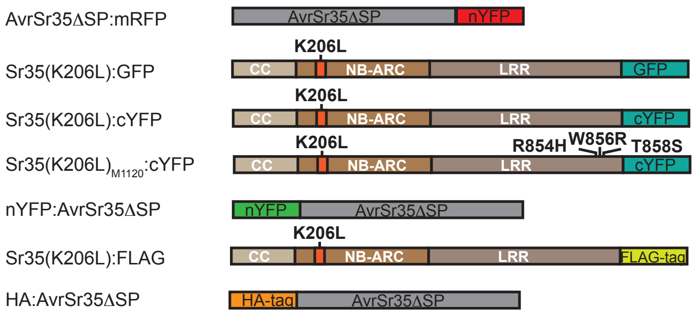

Fig. S8

Schematic representation of constructs used for the subcellular localization, bi-molecular complementation and co-immunoprecipitation analyses shown on Fig. 4.

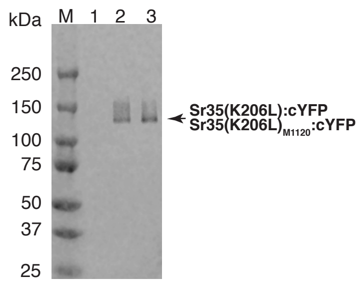

Fig. S9

Immunoblotting of proteins isolated from the non-infiltrated *N. benthamiana* leaves (1) or leaves co-infiltrated with Bi-FC constructs Sr35(K206L):cYFP + nYFP:AvrSr35ΔSP (2), and Sr35(K206L)M1120:cYFP + nYFP:AvrSr35ΔSP (3). The non-infiltrated tobacco plant was used as negative control. The 113 kDa band (lanes 2 and 3) corresponds to Sr35 proteins fused with cYFP. Immunoblotting performed using anti-GFP antibody against cYFP showed that both Sr35 and *Sr35*$_{\mathrm{M1120}}$ protein fusions are stable and expressed at similar levels.

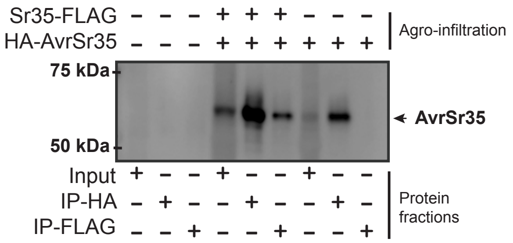

Fig. S10

The immunoprecipitation (IP) of the Sr35 and AvrSr35 proteins tagged with both FLAG and HA epitopes, respectively, and expressed in *N. benthamiana* leaves. Protein complex immunoprecipitations were performed using both anti-HA and anti-FLAG protein isolation columns to obtain IP-HA and IP-FLAG protein fractions. These protein fractions along with the raw protein extracts before column IP (Input protein fraction) were separated in the 10% SDS-PAGE. Immunoblotting was performed using anti-HA antibodies.

Table S1.

A set of randomly selected five EMS mutants of the 99KS76A isolate (race RKQQC) tested on the panel of differential wheat lines carrying different *Sr* genes.

<Provided as separate file: Table S1.docx >

Table S2.

Infection types (IT)* on seedlings using the wild type *Pgt* RKQQC race and two EMS mutants of *Pgt*, M1 and M7, against the differential set of wheat lines carrying different *Sr* genes.

**Set**	**ID**	**Wheat line**	***Sr* gene**	**99KS76 A**	**M1**	**M7**
I	1	ISr5-Ra	5	3+	3+	3+
	2	CnS_T_mono_deriv	21	3+	3+	3+
	3	Vernstine	9e	2	2	2
	4	ISr7b-Ra	7b	3+	3+	3+
II	5	ISr11-Ra	11	2-	2-/3+	2-
	6	ISr6-Ra	6	3+	3+	3+
	7	ISr8a-Ra	8a	3+	3+	3+
	8	CnSr9g	9g	3+	3+	3+
III	9	W2691SrTt-1	36	3+	3+	3+ lif
	10	W2691Sr9b	9b	3+	3+	3+
	11	BtSr30Wst	30	2	2-	2-
	12	Combination VII	17+13	2+	22+	2
IV	13	ISr9a-Ra	9a	3+	3+	3+
	14	ISr9d-Ra	9d	3+	3+	3+
	15	W2691Sr10	10	;1	0;	0;
	16	CnsSrTmp	Tmp	;1	0;	0;
V	17	LcSr24Ag	24	2	22+	22+

	18	Sr31/6*LMPG	31	2-	2	22-
	19	Trident	38	0;	0;	0;
	20	McNair 701	McN	3+	3+	3+
VI	21	Line E	-	3+	3+	3+
	22	Acme	9g	3+	3+	3+
	23	Siouxland	24+31	2-	2	2
	24	Sisson	31+36	2-	2-	NA
VII	25	SwSr22T.B.	22	2	2	2
	26	Agatha/9*LMPG	25	22+	22+	22+
	27	Eagle	26	2	2-	2-
	28	73,214,3-1/9*LMPG	27	0;1/3+	0;1/3+	0;1-/3+
VIII	29	Federation*4/Kavkaz	31	0;	0;	0;
	30	ER 5155	32	2	2	2-
	31	Tetra Canthatch/*Ae. squarrosa*	33	2-	2-	2-
	32	Mq(2)5XG2919	35	0;	2-	2-
IX	33	W3563	37	0;/3+	0;	0;/3+
	34	RL6082	39	-	2-	2-
	35	RL6088	40	2-	2-	2-
	36	TAF 2	44	;2-	2-	;2-
X	37	DAS15	47	;	0;	0;
	38	Satu	Satu	0;	0;	0;
	39	TAM 107-1	1A.1R	12-	;2-	2-
	40	Fed*3/Gabo*51BL.1RS-1-1	R	0;	0;	0;
XI	41	Iumillo	9g+12	0;	0;	0;

	42	Leeds	9e+13	0;	0;	0;
	43	ST464	13	2	2	2
	44	Egypt Na101/6*Marquis	Sr7a	11+	1+3-	31+
XII	45	Steptoe	-	0;13-z	1+3- lif	1+3 lif
	46	Q21861	Rpg1, rpg4, Rpg5	0;	0;1	0;
	47	Morex	Rpg1	0;11+z	12-	11+ lif
	48	QSM20	rpg4/Tpg5	0;	0;1	0;1
	49	DV92	Sr35	0;	3+	3+

*Infection types (IT) at the seedling stage are based on Roelfs and Martens, 1988 (*13*), where IT 0, ;, 1, 2, z, lif or their combinations correspond to low infection types, IT 3 to 4 are considered high infection types, and lif strands for low infection frequency. ITs are refined by adding -, when uredia are somewhat smaller than normal for the infection type, or +, when uredia are somewhat larger than normal for the infection type. Discrete ITs on a single leaf are separated by a comma; range of variation in ITs is shown by indicating the range without comma.

Table S3.

The average size of uredia (pustules) and fungal structure area produced by the *Pgt* mutants and wild-type race RKQQC on the susceptible wheat cultivar Fielder.

	15 days after infection		4 days after infection	
***Pgt* strain**	Mean uredia size (mm^2)	Standard deviation	Mean fungal structure area / infection unit (pixels)	Standard deviation
**M1**	0.024	0.017	28593	11558
**M4**	0.024	0.009	39067	19577
**M7**	0.021	0.012	36941	20676
**Wild-type**	0.035	0.009	22839	19553
ANOVA	F-value = 1.05, df = 3, p-value = 0.41		F-value = 1.09, df = 3, p-value = 0.37	

Table S4.

RNA-seq data generated for the leaves of wheat cultivar Fielder inoculated with wild-type *Pgt* (isolate 99KS76A-1) and its mutants carrying the knock-out mutations in the *AvrSr35* gene (BioProject PRJNA415853).

Sample name	Pgt strain	Time post infection[a]	Biological repl.	Total reads[b]
S_M1-24HPI-rep1	M1	24 HPI	rep1	71493908
S_M1-24HPI-rep2	M1	24 HPI	rep2	73425780
S_M1-24HPI-rep3	M1	24 HPI	rep3	50043948
S_M1-48HPI-rep1	M1	48 HPI	rep1	76434864
S_M1-48HPI-rep2	M1	48 HPI	rep2	45071248
S_M1-48HPI-rep3	M1	48 HPI	rep3	55523420
S_M1-96HPI-rep1	M1	96 HPI	rep1	68284890
S_M1-96HPI-rep2	M1	96 HPI	rep2	78911948
S_M1-96HPI-rep3	M1	96 HPI	rep3	77885124
S_M4-24HPI-rep1	M4	24 HPI	rep1	75294262
S_M4-24HPI-rep2	M4	24 HPI	rep2	43044576
S_M4-24HPI-rep3	M4	24 HPI	rep3	54143310
S_M4-48HPI-rep1	M4	48 HPI	rep1	65469322
S_M4-48HPI-rep2	M4	48 HPI	rep2	65100866
S_M4-48HPI-rep3	M4	48 HPI	rep3	81485452
S_M4-96HPI-rep1	M4	96 HPI	rep1	74296826
S_M4-96HPI-rep2	M4	96 HPI	rep2	76430062
S_M4-96HPI-rep3	M4	96 HPI	rep3	51535330
S_M7-24HPI-rep1	M7	24 HPI	rep1	78192968
S_M7-24HPI-rep2	M7	24 HPI	rep2	62883578
S_M7-24HPI-rep3	M7	24 HPI	rep3	39792544
S_M7-48HPI-rep1	M7	48 HPI	rep1	76163382

S_M7-48HPI-rep2	M7	48 HPI	rep2	71593428
S_M7-48HPI-rep3	M7	48 HPI	rep3	67060948
S_M7-96HPI-rep1	M7	96 HPI	rep1	63657578
S_M7-96HPI-rep2	M7	96 HPI	rep2	78488488
S_M7-96HPI-rep3	M7	96 HPI	rep3	82464872
Mock24H-rep1	Wild type	mock	rep1	57691078
Mock24H-rep2	Wild type	mock	rep2	51077508
Mock24H-rep3	Wild type	mock	rep3	60766166
S_WT-24HPI-rep1	Wild type	24 HPI	rep1	69087510
S_WT-24HPI-rep2	Wild type	24 HPI	rep2	134100980
S_WT-24HPI-rep3	Wild type	24 HPI	rep3	154437332
S_WT-48HPI-rep1	Wild type	48 HPI	rep1	74425614
S_WT-48HPI-rep2	Wild type	48 HPI	rep2	117552574
S_WT-48HPI-rep3	Wild type	48 HPI	rep3	50043336
S_WT-96HPI-rep1	Wild type	96 HPI	rep1	78517386
S_WT-96HPI-rep2	Wild type	96 HPI	rep2	100898188
S_WT-96HPI-rep3	Wild type	96 HPI	rep3	51760526

[a]HPI – hours post inoculation

[b]raw combined single read counts

Table S5.

GO terms showing significant enrichment (FDR ≤ 0.05) in the set of differentially expressed genes between the leaves of mock- and wild-type *Pgt* (isolate 99KS76A-1) inoculated wheat cultivar Fielder.

GO term	GO description	log10 p-value
GO:0002376	immune system process	-6.58
GO:0007623	circadian rhythm	-7.63
GO:0009628	response to abiotic stimulus	-54.00
GO:0015979	Photosynthesis	-99.93
GO:0022613	ribonucleoprotein complex biogenesis	-65.62
GO:0071840	cellular component organization or biogenesis	-11.57
GO:0099131	ATP hydrolysis coupled ion transmembrane transport	-14.35
GO:1901615	organic hydroxy compound metabolic process	-10.36
GO:0006457	protein folding	-24.55
GO:0019684	photosynthesis, light reaction	-56.25
GO:0006801	superoxide metabolic process	-12.83
GO:0009791	post-embryonic development	-11.41
GO:1901135	carbohydrate derivative metabolic process	-32.83
GO:0016311	dephosphorylation	-7.31
GO:0006091	generation of precursor metabolites and energy	-74.68
GO:0051188	cofactor biosynthetic process	-41.51
GO:0051186	cofactor metabolic process	-68.48

GO:0015977	carbon fixation	-7.86
GO:0009853	photorespiration	-9.71
GO:0018298	protein-chromophore linkage	-21.37
GO:0046148	pigment biosynthetic process	-22.69
GO:1901617	organic hydroxy compound biosynthetic process	-8.07
GO:0044255	cellular lipid metabolic process	-18.48
GO:0043094	cellular metabolic compound salvage	-19.12
GO:0008219	cell death	-4.88
GO:0044272	sulfur compound biosynthetic process	-10.36
GO:0044723	single-organism carbohydrate metabolic process	-11.54
GO:0044712	single-organism catabolic process	-14.03
GO:0006081	cellular aldehyde metabolic process	-11.56
GO:1901657	glycosyl compound metabolic process	-10.08
GO:0045454	cell redox homeostasis	-10.57
GO:0031425	chloroplast RNA processing	-8.24
GO:0006629	lipid metabolic process	-9.79
GO:1900865	chloroplast RNA modification	-3.29
GO:0043603	cellular amide metabolic process	-200.67
GO:0030091	protein repair	-7.43
GO:0043069	negative regulation of programmed cell death	-3.56

GO:0072521	purine-containing compound metabolic process	-35.99
GO:0015833	peptide transport	-9.94
GO:0055086	nucleobase-containing small molecule metabolic process	-45.18
GO:0065008	regulation of biological quality	-6.13
GO:0042558	pteridine-containing compound metabolic process	-6.12
GO:0009416	response to light stimulus	-35.62
GO:0006413	translational initiation	-19.36
GO:0006108	malate metabolic process	-3.16
GO:0044281	small molecule metabolic process	-132.46
GO:0046907	intracellular transport	-11.14
GO:0006662	glycerol ether metabolic process	-8.23
GO:0018904	ether metabolic process	-8.23
GO:0006818	hydrogen transport	-6.31
GO:0070084	protein initiator methionine removal	-4.65
GO:0006575	cellular modified amino acid metabolic process	-6.18
GO:0033013	tetrapyrrole metabolic process	-31.05
GO:0051707	response to other organism	-10.33
GO:0010035	response to inorganic substance	-26.26
GO:0034599	cellular response to oxidative stress	-18.31

GO:0034614	cellular response to reactive oxygen species	-12.64
GO:0006955	immune response	-4.91
GO:0009607	response to biotic stimulus	-7.40
GO:0051641	cellular localization	-12.18
GO:0071705	nitrogen compound transport	-6.90
GO:0048584	positive regulation of response to stimulus	-4.69
GO:0072524	pyridine-containing compound metabolic process	-17.57
GO:0009657	plastid organization	-62.90
GO:0033036	macromolecule localization	-4.24
GO:0042180	cellular ketone metabolic process	-7.25
GO:0009658	chloroplast organization	-40.90
GO:0044711	single-organism biosynthetic process	-69.94
GO:0016072	rRNA metabolic process	-41.47
GO:0010190	cytochrome b6f complex assembly	-3.67
GO:0009605	response to external stimulus	-16.48
GO:0031349	positive regulation of defense response	-4.86
GO:0080134	regulation of response to stress	-3.71

Table S6.

Summary of sequence data used for assembling the genome of *Pgt* isolate 99KS76A-1 (RKQQC race).

Library	Sequencer	Average read length	Number of reads	Number of bases[a]
1	Illumina MiSeq	296	62,781,618	18,559,989,584
2	Roche 454	391	1,426,527	558,467,858
3	PacBio	2,349	264,532	621,404,037
Total				19,739,861,479

a - after quality trimming

Table S7.

Summary of Illumina sequence data generated for 15 EMS mutant strains of *Pgt*.

***Pgt* mutants**	**Raw reads**	**Reads passed QC**	**Mapped reads**
M1	82,091,932	76,118,455	58,326,583
M2	51,290,470	49,619,008	40,584,633
M3	84,949,786	80,247,091	55,761,680
M4	105,932,340	102,107,182	91,379,253
M5	153,797,390	146,680,099	95,642,043
M6	98,706,490	94,613,226	85,727,717
M7	131,754,854	124,808,099	102,960,385
M8	109,674,738	103,600,923	80,610,560
M9	106,560,764	101,214,023	91,079,974
M10	120,434,156	113,715,743	92,048,840
M11	77,091,268	72,917,378	61,716,667
M12	46,914,984	45,126,833	34,956,847
M13	53,203,268	51,272,436	21,630,040
M14	48,311,656	46,694,537	28,372,655
M15	56,325,312	54,508,028	46,174,679
Average	88,469,294	84,216,204	65,798,170

Table S8.

The summary of EMS-induced mutations discovered in each of the 15 mutant strains and their functional effect based on the analysis using the SnpEff program.

	SNP effects[a]											
Mutants	DOWNSTREAM	INTERGENIC	INTRON	NON_SYNONYMOUS	START_GAINED	STOP_GAINED	SYNONYMOUS_CODING	SYNONYMOUS_STOP	UPSTREAM	UTR_3_PRIME	UTR_5_PRIME	Total[b]
M1	50	30	1	4	0	0	5	0	3	1	0	94
M2	782	961	43	130	2	10	54	0	105	8	5	2100
M3	397	575	22	71	1	3	26	0	70	5	2	1172
M4	112	198	4	17	0	2	5	0	15	1	0	354
M5	744	896	36	129	3	11	48	1	116	10	8	2002
M6	129	167	8	18	1	1	10	0	18	1	11	364
M7	555	812	32	107	0	10	45	0	79	2	3	1645
M8	6814	2444	95	1967	49	149	3817	13	225	8	12	15593
M9	556	727	31	88	3	7	44	0	69	12	11	1548
M10	818	1008	44	106	2	12	75	0	118	3	6	2192
M11	371	506	21	55	0	3	39	0	62	4	2	1063
M12	237	273	14	42	0	4	20	0	23	1	1	615
M13	571	537	36	81	2	7	58	0	80	1	5	1378
M14	1559	1540	78	248	2	14	224	0	168	16	5	3854
M15	21	29	3	6	1	4	3	0	9	1	2	79

a - SNP effects are assessed according to the SnpEff program.

b - Low number of mutations likely caused by inefficient EMS treatment.

Table S9.

The positions of EMS mutations in the candidate *AvrSr35* gene (scaffold 2351) identified by analyzing NGS and Sanger sequencing data (0 – wild-type base, 1 – mutant base).

Positions: scaff/gene	Wil/ Mut.	M1	M2	M3	M4	M5	M6	M7	M8	M9	M10	M11	M12	M13	M14	M15	Effect
1843/411	C/T	0	0	0	0	0	0	0	0	0	0	**1**	0	0	0	0	STOP
2009/577	C/T	0	0	0	0	**1**	0	0	0	0	0	0	0	0	0	0	STOP
2206/774	G/A	**1**	0	0	0	0	**1**	0	0	0	0	0	0	0	0	0	NS
2460/1028	C/T	0	0	0	0	0	0	0	0	0	0	0	**1**	0	0	0	STOP
2546/1114	G/A	0	0	0	0	0	0	0	0	0	0	0	0	0	0	**1**	STOP
2909/1477	G/A	0	0	0	0	0	0	**1**	0	0	0	0	0	0	0	0	STOP
2941/1509	G/A	0	0	**1**	0	0	0	0	0	0	0	0	0	0	0	0	SPL.
3096/1664	G/A	0	**1**	0	0	0	0	0	0	0	0	0	0	0	**1**	0	STOP
3161/1729	C/T	0	0	0	0	0	0	0	0	0	0	0	0	**1**	0	0	STOP
3287/1855	C/T	0	0	0	**1**	0	0	0	**1**	**1**	0	0	0	0	0	0	STOP
3641/2209	C/T	0	0	0	0	0	0	0	0	0	**1**	0	0	0	0	0	STOP

Table S10.

List of primers used in the study (provided as a separate file).

Table S11.

The list of *Pgt* isolates for studying the genetic diversity of the *AvrSr35* gene candidate.

Pgt isolate	Location	Year	Race[a]	Virulence on *Sr35* gene[b]
75WA1652A	Washington, USA	1975	QCCSM	Virulent
59KS19	Kansas, USA	1959	MCCFC	Virulent
76WA1358C	Washington, USA	1976	SMLLM	Virulent
09CA115-2	California, USA	2009	BCCBC	Virulent
75WA1652	Washington, USA	1975	QCCSM	Virulent
01SD80A	South Dakota, USA	2001	QCCJB	Virulent
09ID073-2	Idaho, USA	1009	SCCSC	Virulent
76WA1397B	Washington, USA	1976	QFBDC	Virulent
74WA1331B	Washington, USA	1974	BFBJC	Virulent
69MN399	Minnesota, USA	1969	QTHJC	Virulent
06ND76C	North Dakota, USA	2006	QFCSC	Virulent
76WA1204C	Washington, USA	1976	SBCSC	Virulent
74MN1049	Minnesota, USA	1974	TPMKC	Avirulent[c]
75-36-700-3	Pennsylvania, USA	1975	SCCLC	Avirulent[c]
04KEN156/04	Kenya	2004	TTKSK	Avirulent
84KEN654B	Kenya	1984	R_TTF	Avirulent
01MN84A-1-2	Minnesota, USA	1984	TTTTF	Avirulent
99KS76A_1	Kansas, USA	1999	RKQQC	Avirulent[c]
77ND82A	North Dakota, USA	1977	RCRSC	Avirulent[c]
72CA1A	California, USA	1972	TPLK	Avirulent[c]
69SD657C	South Dakota, USA	1969	RHTS	Avirulent[d]

61PA80A	Pennsylvania, USA	1961	RKLQ	Avirulent
06KEN19-V-3	Kenya	2006	TTKST	Avirulent
Uv59.4	South Africa	2007	TTKSP	Avirulent
07KEN24-4	Kenya	2007	TTTSK	Avirulent
Uv55.2	South Africa	2009	TTKSF	Avirulent
00MN99c	Minnesota, USA	2000	RCRSC	Avirulent

[a] Based on North American nomenclature (*29*).

[b] Isolates were tested for virulence on the wheat line 4071 carrying the *Sr35* gene. Isolates were considered virulent if they showed scores from 2 to 4; isolates were classified as avirulent if they had scores 0 or 0; (*30*).

[c] Two alleles in Fig. 2B.

[d] Three gene variants in Fig. 2B.

Table S12.

Plasmid constructs used in the study.

Construct Name	Experiment	Vector backbone	Antibiotic	Insert description
pGEMT-cDNA-AvrSr35	Cloning of RT-PCR product	pGEM-T	Ampicillin	AvrSr35 (AA1-578)
pAvrSr35-ORF-TDNA	*N. benthamiana* HR assay	pIPKb004 (Gateway destination vector)	Streptomycin	CaMV 35S - AvrSr35 (AA1-578)
pAvrSr35-ΔSP-TDNA	*N. benthamiana* HR assay	pIPKb004 (Gateway destination vector)	Streptomycin	CaMV 35S - AvrSr35 (AA27-578)
pAvrSr35(Q72*)-ΔSP	*N. benthamiana* HR assay	pIPKb004 (Gateway destination vector)	Streptomycin	CaMV 35S - AvrSr35(AA27-71)
pSr35-TDNA	*N. benthamiana* HR assay	pIPKb004 (Gateway destination vector)	Streptomycin	CaMV 35S - Sr35 (AA1-919)
pSr35(K206L)-TDNA	*N. benthamiana* HR assay	pIPKb004 (Gateway destination vector)	Streptomycin	CaMV 35S - Sr35 (AA1-919): p.K206L
pSr35v1120-TDNA	*N. benthamiana* HR assay	pIPKb004 (Gateway destination vector)	Streptomycin	Sr35(AA1-919): p.R854H+W856R+T858S
pAvrSr35-mRFP	Sub-cellular localization	pSITE-4NA (Gateway Destination vector)	Kanamycin	AvrSr35(AA1-578) C-terminal fusion of mRFP CDS
pAvrSr35-ΔSP-mRFP	Sub-cellular localization	pSITE-4NA (Gateway Destination vector)	Kanamycin	Fusion of AvrSr35(AA27-578) to the C-terminal of mRFP CDS
RIN4-GFP	Plasma membrane marker		Kanamycin	Fusion of GFP with RIN4
ER-mCherry	ER marker	pBIN20	Kanamycin	C-terminal fusion of HDEL and AtWAK2 signal peptide to mCherry
pSr35(K206L)-eGFP	Sub-cellular localization	pIPKb004 (Gateway destination vector)	Kanamycin	Fusion of CaMV 35S - Sr35(AA1-919): p.K206L; C-terminal fusion with eGFP
pSr35(K206L)-cYFP	BiFC	pIPKb004 (Gateway destination vector)	Streptomycin	Fusion of CaMV 35S - Sr35(AA1-919): p.K206L to the C-terminal domain of eYFP (AA155-239)

pnYFP-AvrSr35-ΔSP	BiFC	pIPKb004 (Gateway destination vector)	Streptomycin	CaMV 35S - AvrSr35(AA27-578) N-terminal fusion with eYFP (AA1-154)
pSr35(K206L)$_{M1120}$-cYFP	BiFC	pIPKb004 (Gateway destination vector)	Streptomycin	CaMV 35S - Sr35(AA1-919):p.K206L+R854H+W856R+T858S to the C-terminal domain of eYFP (AA155-239)
pnYFP-AvrSr35(Q72*)-ΔSP	BiFC	pIPKb004 (Gateway destination vector)	Streptomycin	CaMV 35S - AvrSr35(AA27-72) N-terminal fusion with eYFP (AA1-154)
pET-SUMO-AvrSr35-ΔSP	Protein expression	pET-SUMO (TA cloning)	Kanamycin	AvrSr35 (AA27-578)
pAvrSr35-ΔSP-TDNA c-HA	CoIP	pIPKb004 (Gateway destination vector)	Streptomycin	CaMV 35S – HA-AvrSr35 (AA27-578)
pSr35-TDNA-n-Flag	CoIP	pIPKb004 (Gateway destination vector)	Streptomycin	CaMV 35S - Sr35 (AA1-919)-Flag

Movie S1 (separate file)

Time-lapse imaging of tobacco leaves co-infiltrated with the combinations of wild-type and mutant *Sr35* and *AvrSr35* constructs. The layout of agro-infiltrated constructs corresponds to that presented on Figs. 3C and 3D. The images were taken every 15 min over a period of 48 hours after inoculation.

Dataset S1 (separate file)

EMS induced mutations discovered in 15 *Pgt* mutant strains of 99KS76A-1 isolate and their effects assessed using SnpEff program.

Dataset S2 (separate file)

The sequences of the *AvrSr35* gene for *Sr35*-virulent and *Sr35*-avirulent *Pgt* isolates.

References

1. Z. A. Pretorius, R. P. Singh, W. W. Wagoire, T. S. Payne, Detection of virulence to wheat stem rust resistance gene *Sr31* in *Puccinia graminis* f. sp. *tritici* in Uganda. *Plant Dis.* **84**, 203 (2000). doi:10.1094/PDIS.2000.84.2.203B

2. R. P. Singh, D. P. Hodson, Y. Jin, E. S. Lagudah, M. A. Ayliffe, S. Bhavani, M. N. Rouse, Z. A. Pretorius, L. J. Szabo, J. Huerta-Espino, B. R. Basnet, C. Lan, M. S. Hovmøller, Emergence and spread of new races of wheat stem rust fungus: Continued threat to food security and prospects of genetic control. *Phytopathology* **105**, 872–884 (2015). doi:10.1094/PHYTO-01-15-0030-FI Medline

3. J. D. G. Jones, J. L. Dangl, The plant immune system. *Nature* **444**, 323–329 (2006). doi:10.1038/nature05286 Medline

4. J. G. Ellis, M. Rafiqi, P. Gan, A. Chakrabarti, P. N. Dodds, Recent progress in discovery and functional analysis of effector proteins of fungal and oomycete plant pathogens. *Curr. Opin. Plant Biol.* **12**, 399–405 (2009). doi:10.1016/j.pbi.2009.05.004 Medline

5. J. M. Elmore, Z.-J. D. Lin, G. Coaker, Plant NB-LRR signaling: Upstreams and downstreams. *Curr. Opin. Plant Biol.* **14**, 365–371 (2011). doi:10.1016/j.pbi.2011.03.011 Medline

6. C. Saintenac, W. Zhang, A. Salcedo, M. N. Rouse, H. N. Trick, E. Akhunov, J. Dubcovsky, Identification of wheat gene Sr35 that confers resistance to Ug99 stem rust race group. *Science* **341**, 783–786 (2013). doi:10.1126/science.1239022 Medline

7. Materials and methods are available as supplementary materials.

8. W. B. Rutter, A. Salcedo, A. Akhunova, F. He, S. Wang, H. Liang, R. L. Bowden, E. Akhunov, Divergent and convergent modes of interaction between wheat and *Puccinia graminis* f. sp. *tritici* isolates revealed by the comparative gene co-expression network and genome analyses. *BMC Genomics* **18**, 291 (2017). doi:10.1186/s12864-017-3678-6 Medline

9. S. Duplessis, C. A. Cuomo, Y.-C. Lin, A. Aerts, E. Tisserant, C. Veneault-Fourrey, D. L. Joly, S. Hacquard, J. Amselem, B. L. Cantarel, R. Chiu, P. M. Coutinho, N. Feau, M. Field, P. Frey, E. Gelhaye, J. Goldberg, M. G. Grabherr, C. D. Kodira, A. Kohler, U. Kües, E. A. Lindquist, S. M. Lucas, R. Mago, E. Mauceli, E. Morin, C. Murat, J. L. Pangilinan, R. Park, M. Pearson, H. Quesneville, N. Rouhier, S. Sakthikumar, A. A. Salamov, J. Schmutz, B. Selles, H. Shapiro, P. Tanguay, G. A. Tuskan, B. Henrissat, Y. Van de Peer, P. Rouzé, J. G. Ellis, P. N. Dodds, J. E. Schein, S. Zhong, R. C. Hamelin, I. V. Grigoriev, L. J. Szabo, F. Martin, Obligate biotrophy features unraveled by the genomic analysis of rust fungi. *Proc. Natl. Acad. Sci. U.S.A.* **108**, 9166–9171 (2011). doi:10.1073/pnas.1019315108 Medline

10. D. Cantu, V. Segovia, D. MacLean, R. Bayles, X. Chen, S. Kamoun, J. Dubcovsky, D. G. O. Saunders, C. Uauy, Genome analyses of the wheat yellow (stripe) rust pathogen *Puccinia striiformis* f. sp. *tritici* reveal polymorphic and haustorial expressed secreted proteins as candidate effectors. *BMC Genomics* **14**, 270 (2013). doi:10.1186/1471-2164-14-270 Medline

11. J. Chen, N. M. Upadhyaya, D. Ortiz, J. Sperschneider, F. Li, C. Bouton, S. Breen, C. Dong, B. Xu, X. Zhang, R. Mago, K. Newell, X. Xia, M. Bernoux, J. M. Taylor, B. Steffenson,

Y. Jin, P. Zhang, K. Kanyuka, M. Figueroa, J. G. Ellis, R. F. Park, P. N. Dodds, Loss of *AvrSr50* by somatic exchange in stem rust leads to virulence for *Sr50* resistance in wheat. *Science* **358**, 1607–1610 (2017). doi:10.1126/science.aao4810

12. M. Ayliffe, R. Devilla, R. Mago, R. White, M. Talbot, A. Pryor, H. Leung, Nonhost resistance of rice to rust pathogens. *Mol. Plant. Microbe Interact.* **24**, 1143–1155 (2011). doi:10.1094/MPMI-04-11-0100 Medline

13. A. P. Roelfs, J. W. Martens, An international system of nomenclature for *Puccinia graminis* f. sp. *tritici*. *Phytopathology* **78**, 526–533 (1988). doi:10.1094/Phyto-78-526

14. B. J. Clavijo, L. Venturini, C. Schudoma, G. G. Accinelli, G. Kaithakottil, J. Wright, P. Borrill, G. Kettleborough, D. Heavens, H. Chapman, J. Lipscombe, T. Barker, F.-H. Lu, N. McKenzie, D. Raats, R. H. Ramirez-Gonzalez, A. Coince, N. Peel, L. Percival-Alwyn, O. Duncan, J. Trösch, G. Yu, D. M. Bolser, G. Namaati, A. Kerhornou, M. Spannagl, H. Gundlach, G. Haberer, R. P. Davey, C. Fosker, F. D. Palma, A. L. Phillips, A. H. Millar, P. J. Kersey, C. Uauy, K. V. Krasileva, D. Swarbreck, M. W. Bevan, M. D. Clark, An improved assembly and annotation of the allohexaploid wheat genome identifies complete families of agronomic genes and provides genomic evidence for chromosomal translocations. *Genome Res.* **27**, 885–896 (2017). doi:10.1101/gr.217117.116 Medline

15. N. L. Bray, H. Pimentel, P. Melsted, L. Pachter, Near-optimal probabilistic RNA-seq quantification. *Nat. Biotechnol.* **34**, 525–527 (2016). doi:10.1038/nbt.3519 Medline

16. A. Dobon, D. C. E. Bunting, L. E. Cabrera-Quio, C. Uauy, D. G. Saunders, The host-pathogen interaction between wheat and yellow rust induces temporally coordinated waves of gene expression. *BMC Genomics* **17**, 380 (2016). doi:10.1186/s12864-016-2684-4 Medline

17. W. Truman, M. T. de Zabala, M. Grant, Type III effectors orchestrate a complex interplay between transcriptional networks to modify basal defence responses during pathogenesis and resistance. *Plant J.* **46**, 14–33 (2006). doi:10.1111/j.1365-313X.2006.02672.x Medline

18. M. G. Grabherr, B. J. Haas, M. Yassour, J. Z. Levin, D. A. Thompson, I. Amit, X. Adiconis, L. Fan, R. Raychowdhury, Q. Zeng, Z. Chen, E. Mauceli, N. Hacohen, A. Gnirke, N. Rhind, F. di Palma, B. W. Birren, C. Nusbaum, K. Lindblad-Toh, N. Friedman, A. Regev, Full-length transcriptome assembly from RNA-seq data without a reference genome. *Nat. Biotechnol.* **29**, 644–652 (2011). doi:10.1038/nbt.1883 Medline

19. B. J. Haas, Q. Zeng, M. D. Pearson, C. A. Cuomo, J. R. Wortman, Approaches to fungal genome annotation. *Mycology* **2**, 118–141 (2011). Medline

20. F. A. Simão, R. M. Waterhouse, P. Ioannidis, E. V. Kriventseva, E. M. Zdobnov, BUSCO: Assessing genome assembly and annotation completeness with single-copy orthologs. *Bioinformatics* **31**, 3210–3212 (2015). Medline

21. A. McKenna, M. Hanna, E. Banks, A. Sivachenko, K. Cibulskis, A. Kernytsky, K. Garimella, D. Altshuler, S. Gabriel, M. Daly, M. A. DePristo, The Genome Analysis Toolkit: A MapReduce framework for analyzing next-generation DNA sequencing data. *Genome Res.* **20**, 1297–1303 (2010). doi:10.1101/gr.107524.110 Medline

22. I. M. Henry, U. Nagalakshmi, M. C. Lieberman, K. J. Ngo, K. V. Krasileva, H. Vasquez-Gross, A. Akhunova, E. Akhunov, J. Dubcovsky, T. H. Tai, L. Comai, Efficient genome-wide detection and cataloging of EMS-induced mutations using exome capture and next-generation sequencing. *Plant Cell* **26**, 1382–1397 (2014). doi:10.1105/tpc.113.121590 Medline

23. P. N. Dodds, G. J. Lawrence, A.-M. Catanzariti, T. Teh, C.-I. A. Wang, M. A. Ayliffe, B. Kobe, J. G. Ellis, Direct protein interaction underlies gene-for-gene specificity and coevolution of the flax resistance genes and flax rust avirulence genes. *Proc. Natl. Acad. Sci. U.S.A.* **103**, 8888–8893 (2006). doi:10.1073/pnas.0602577103 Medline

24. J. Yang, R. Yan, A. Roy, D. Xu, J. Poisson, Y. Zhang, The I-TASSER Suite: Protein structure and function prediction. *Nat. Methods* **12**, 7–8 (2015). doi:10.1038/nmeth.3213 Medline

25. W. I. L. Tameling, J. H. Vossen, M. Albrecht, T. Lengauer, J. A. Berden, M. A. Haring, B. J. Cornelissen, F. L. Takken, Mutations in the NB-ARC domain of I-2 that impair ATP hydrolysis cause autoactivation. *Plant Physiol.* **140**, 1233–1245 (2006). doi:10.1104/pp.105.073510 Medline

26. B. K. Nelson, X. Cai, A. Nebenführ, A multicolored set of in vivo organelle markers for co-localization studies in *Arabidopsis* and other plants. *Plant J.* **51**, 1126–1136 (2007). doi:10.1111/j.1365-313X.2007.03212.x Medline

27. D. Mackey, Y. Belkhadir, J. M. Alonso, J. R. Ecker, J. L. Dangl, *Arabidopsis* RIN4 is a target of the type III virulence effector AvrRpt2 and modulates RPS2-mediated resistance. *Cell* **112**, 379–389 (2003). doi:10.1016/S0092-8674(03)00040-0 Medline

28. O. Emanuelsson, S. Brunak, G. von Heijne, H. Nielsen, Locating proteins in the cell using TargetP, SignalP and related tools. *Nat. Protoc.* **2**, 953–971 (2007). doi:10.1038/nprot.2007.131 Medline

29. Y. Jin, L. J. Szabo, Z. A. Pretorius, R. P. Singh, R. Ward, T. Fetch Jr., Detection of virulence to resistance gene Sr24 within race TTKS of *Puccinia graminis* f. sp. *tritici*. *Plant Dis.* **92**, 923–926 (2008). doi:10.1094/PDIS-92-6-0923

30. E. Stakman, D. Stewart, W. Loegering, *Identification of Physiologic Races of* Puccinia graminis *var.* tritici (U.S. Department of Agriculture, Agricultural Research Service, 1962).
