# Supplementary material for: Variation in the AvrSr35 gene determines Sr35 resistance against wheat stem rust race Ug99
Source: Science. 2017 Dec 22;358(6370):1604–6. doi: 10.1126/science.aao7294 (PMC6518949; doi:10.1126/science.aao7294)
Supplement: Supplementary file 2 [file Science-358-1604-s2.docx]

Table S1.

A set of randomly selected five EMS mutants of the 99KS76A isolate (race RKQQC) screened for infection types (IT)* on the panel of mono-genic differential wheat lines carrying different *Sr* genes.

| Differential# | Differentials | Sr genes | 99KS76A | | M1 | | M7 | | M6 | | M9 | | M11 | | RACE |
| --- | --- | --- | --- | --- | --- | --- | --- | --- | --- | --- | --- | --- | --- | --- | --- |
|  |  |  | Scoring | IT | Scoring | IT | Scoring | IT | Scoring | IT | Scoring | IT | Scoring | IT |  |
| 1 | ISr5-Ra | **5** | 3+ | H | 4 | H | 3 | H | 4 | H | 4 | H | 0 | H | R |
| 2 | CnS_T_mono_deriv | **21** | 3+ | H | 4 | H | 4 | H | 4 | H | 4 | H | X | H |  |
| 3 | Vernstine | **9e** | 2 | L | 2 | L | 2 | L | 0 | L | 2 | L | 0 | L |  |
| 4 | ISr7b-Ra | **7b** | 3+ | H | 4 | H | 3 | H | 3 | H | 3,4 | H | 0 | L |  |
| 5 | ISr11-Ra | **11** | 2- | L | 1 | L | 2 | L | 1 | L | 4 | L | 0 | L | K |
| 6 | ISr6-Ra | **6** | 3+ | H | 4 | H | 3,4 | H | 3 | H | 3,4 | H | 4 | H |  |
| 7 | ISr8a-Ra | **8a** | 3+ | H | 4 | H | 4 | H | 3,4 | H | 4 | H | 4 | H |  |
| 8 | CnSr9g | **9g** | 3+ | H | 4 | H | 4 | H | 4 | H | 4 | H | 4 | H |  |
| 9 | W2691SrTt-1 | **36** | 3+ | H | 4 | H | 4 | H | 3,4 | H | X | H | ;,1 | H | Q |
| 10 | W2691Sr9b | **9b** | 3+ | H | 4 | H | 3 | H | 4 | H | 4 | H | 3 | H |  |
| 11 | BtSr30Wst | **30** | 2 | L | 2 | L | 2 | L | 1 | L | 2 | L | 0 | L |  |
| 12 | Combination VII | **17** | 2+ | L | X | L | 1,2 | L | ; | L | 1,2 | L | 2 | L |  |
| 13 | ISr9a-Ra | **9a** | 3+ | H | 4 | H | 3+ | H | 4 | H | 4 | H | 4 | H | Q |
| 14 | ISr9d-Ra | **9d** | 3+ | H | 3,4 | H | 4 | H | 4 | H | 4 | H | 3+ | H |  |
| 15 | W2691Sr10 | **10** | ;1 | L | 2 | L | 2+ | L | 2 | L | ;1 | L | ;1 | L |  |
| 16 | CnsSrTmp | **Tmp** | ;1 | L | ;1 | L | 2+ | L | 2 | L | 2+ | L | ;1 | L |  |
| 17 | LcSr24Ag | **24** | 2 | L | 2,3 | L | 2 | L | 3 | L | 3 | L | 2 | L | C |
| 18 | Sr31/6*LMPG | **31** | 2- | L | 2+ | L | 2- | L | 2 | L | 2 | L | 3 | L |  |
| 19 | Trident or VPM1? | **38** | 0; | L | 0 | L | 0 | L | ; | L | 1 | L | ; | L |  |
| 20 | McNair 701 | **McN** | 3+ | H | 4 | H | 4 | H | 4 | H | 4 | H | 4 | H |  |
|  | G21919 | **35** | 0; | L | 3+ | H | 3+ | H | 4 | H | 4 | H | 4 | H |  |
|  | Morocco | **Susceptible** | 3+ | H | 4 | H | 4 | H | 4 | H | 4 | H | 3 | H |  |

*Infection types (IT) at the seedling stage are based on Roelfs and Martens, 1988 *(13)*, where IT 0, ;, 1, 2, z, lif or their combinations correspond to low infection types, IT 3 to 4 are considered high infection types, and lif strands for low infection frequency. ITs are refined by adding -, when uredia are somewhat smaller than normal for the infection type, or +, when uredia are somewhat larger than normal for the infection type. Discrete ITs on a single leaf are separated by a comma; range of variation in ITs is shown by indicating the range without comma.
